# Drought adaptation index (DAI) based on BLUP as a selection approach for drought-resilient switchgrass germplasm

**DOI:** 10.3389/fgene.2025.1626083

**Published:** 2025-08-25

**Authors:** Shiva Om Makaju, Hari Bahadur Chhetri, Chanaka Roshan Abeyratne, Mirko Pavicic, Hari Poudel, Jazib Ali Irfan, Anita Giabardo, Katrien M. Devos, Daniel Jacobson, Ali Mekki Missaoui

**Affiliations:** 1 Center for Applied Genetic Technologies, University of Georgia, Athens, GA, United States; 2 Institute of Plant Breeding, Genetics and Genomics, University of Georgia, Athens, GA, United States; 3 Department of Crop and Soil Sciences, University of Georgia, Athens, GA, United States; 4 Biosciences Division, Oak Ridge National Laboratory, Oak Ridge, TN, United States; 5 Agriculture and Agri‐Food Canada, Lethbridge Research and Development Centre, Lethbridge, AB, Canada; 6 Department of Plant Biology, University of Georgia, Athens, GA, United States

**Keywords:** switchgrass, bioenergy, drought adaptation, yield stability, BLUP, drought adaptation index, stress tolerance indices, biomass

## Abstract

This study introduces a Drought Adaptation Index (DAI), derived from Best Linear Unbiased Prediction (BLUP), as a method to assess drought resilience in switchgrass (*Panicum virgatum* L.). A panel of 404 genotypes was evaluated under drought-stressed (CV) and well-watered (UC) conditions over four consecutive years (2019–2022). BLUP-estimated biomass yields were used to calculate the DAI, which enabled classification of genotypes into four adaptation groups: very well-adapted, well-adapted, adapted, and unadapted. The DAI was compared with conventional drought tolerance indices, including the Stress Susceptibility Index (SSI), Stress Tolerance Index (STI), Geometric Mean Productivity (GMP), and Yield Stability Index (YSI). Correlation analyses demonstrated strong agreement between DAI and these indices, supporting its validity and consistency. Biplot analyses using the Genotype plus Genotype-by-Environment Interaction (GGE) and Additive Main Effects and Multiplicative Interaction (AMMI) models revealed significant genotype-by-environment interactions (GEI) and identified J222.A, J463.A, and J295.A. A as high-performing genotypes, with J222.A exhibiting greater yield stability across treatments and years. Additionally, DAI isoline curves provided a graphical representation of differential genotype performance under drought and control conditions. These visualizations aided in distinguishing genotypes with stable and superior biomass yield across contrasting environments. Overall, the BLUP-based DAI is a robust and practical selection tool that improves the accuracy of identifying drought-resilient, high-yielding switchgrass genotypes. Its integration into breeding programs offers a comprehensive framework for improving biomass productivity and stress adaptation under variable climatic conditions. The application of DAI supports the development of climate-resilient cultivars and contributes to sustainable bioenergy and forage production systems.

## 1 Introduction

Switchgrass (*Panicum virgatum* L.) is a warm-season, perennial C4 grass with dual use as bioenergy feedstock and forage for livestock. Its adaptability to a wide range of environmental conditions, across North America, makes it a valuable species for sustainable biomass production and ecological conservation (Perlack et al., 2011). In addition to its bioenergy potential and forage use, switchgrass plays a vital role in soil and water conservation due to its extensive root system and ability to thrive in marginal lands (Parrish and Fike, 2005).

Switchgrass exhibits substantial genetic and phenotypic diversity and is subdivided into three ecotypes: upland, lowland, and coastal (Lovell et al., 2021). These ecotypes differ in morphology, growth habits, and adaptation to environmental conditions. Lowland ecotypes generally exhibit higher biomass productivity, are predominantly tetraploid, and thrive in warmer, wetter environments (Fike et al., 2006; Perlack et al., 2011). Upland ecotypes, which can be either tetraploid or octaploid, are adapted to lower precipitation, cooler climates, and typically have thinner tillers and narrower leaves (Fike et al., 2006; Perlack et al., 2011). Coastal ecotypes display an intermediate combination of traits, possessing a lowland-like plant structure but upland-like leaf characteristics (Lovell et al., 2021). This classification is largely based on their geographical origins and ecological adaptations, with upland ecotypes favoring drier, elevated habitats and lowlands preferring riparian zones and floodplains (Casler et al., 2011). While tetraploids are predominant in lowland ecotypes, both tetraploids and octaploids are found in upland ecotypes. Switchgrass follows a disomic inheritance mode (Okada et al., 2010), and the genome size of tetraploid switchgrass is estimated to be 1,130 Mb (Lovell et al., 2021). Identifying superior performance and selecting consistent genotypes are critical for the success of crop improvement programs.

Drought stress is one of the most critical environmental factors limiting biomass yield and stand persistence in switchgrass for multiple years. The ability of switchgrass genotypes to maintain high yield under drought conditions varies significantly, necessitating robust selection strategies that integrate phenotypic performance with genetic resilience. Genotype plus Genotype-by-Environment Interaction (GGE) biplot analysis has been widely used to study genotype-by-environment interactions (GEI) and the stability of genotypes in various crops. Missaoui et al. (2023) applied this method to evaluate tall fescue experimental populations selected under grazing pressure in stress environments. Their study assessed ten experimental populations and six standard checks across nine environments, revealing significant variations in yield due to populations, locations, years, and GEI. While lowland switchgrass germplasm typically yield higher biomass under optimal conditions, upland genotypes often exhibit superior drought tolerance due to their adaptation to arid environments (Casler et al., 2011).

Switchgrass breeding programs must consider its complex reproductive biology. As a wind-pollinated, outcrossing species with allopolyploidy, switchgrass exhibits tetraploids and octaploids as the most common ploidy levels, which adds to the complexity of breeding efforts. Traditionally, breeding programs for switchgrass improvement have relied on recurrent selection methods, such as half-sib family selection, among- and within-family selection, and progeny testing, to enhance desirable traits (Casler and Brummer, 2008). These selection approaches involve evaluating genotypes over multiple years, selecting the best-performing genotypes, and conducting polycrosses for genetic improvement. While effective, these methods are time-consuming, requiring multiple selection cycles to achieve significant genetic gains. Maximizing genetic gains depends on the time required to complete a selection cycle and the selection accuracy, often measured using Pearson’s correlation coefficient.

With advances in genomic selection, breeders can now predict genetic values based on genome-wide markers, accelerating genetic gains (Meuwissen et al., 2001). Genomic selection is particularly advantageous for complex traits controlled by multiple quantitative trait loci (QTL), such as biomass yield and drought tolerance (Bernardo, 2008). When DNA sequence information is available, the development of genomic prediction models reduces the reliance on costly phenotyping in subsequent selection cycles. However, in the absence of extensive genomic data, phenotypic indices remain crucial for assessing stress tolerance and yield stability across diverse environments. Best Linear Unbiased Prediction (BLUP) is a widely adopted statistical approach in plant breeding that enables precise genetic evaluation by accounting for environmental and spatial variation in field trials. BLUP estimates genetic values more accurately than raw phenotypic data (Viana et al., 2022), improving selection efficiency for stress-adapted genotypes. Piepho et al. (2008) provided a comprehensive review of BLUP and its applications in plant breeding and variety testing, emphasizing its shrinkage property, which enhances accuracy by reducing errors. The review explored its applications in plant breeding, both with and without known parentage, including single trials improved by spatial models, multi-environment trials where BLUP outperformed BLUE for estimating main effects, and spatial models with flexible variance structures. Despite these advancements, drought selection methods often fail to simultaneously capture high yield and drought resilience, necessitating improved selection approaches.

Various drought adaptation indices have been used to assess crop performance across various species. Sánchez-Reinoso et al. (2020) evaluated the effectiveness of nine drought tolerance indices - Stress Susceptibility Index (SSI), Tolerance (TOL), Mean Productivity (MP), Geometric Mean Productivity (GMP), Stress Tolerance Index (STI), Yield Stability Index (YSI), Yield Index (YI), Harmonic Mean (HM), and Drought Sensitivity Index (DSI) - in identifying superior common bean genotypes under water deficit during vegetative and reproductive stages. Their study found that SSI was the most effective in distinguishing genotypes least affected by drought. Aydınşakir et al. (2025) assessed drought tolerance of four sorghum genotypes (Uzun, Erdurmuş, Beydarı, and Ogretmenoglu) in Antalya, Turkiye, under full and deficit irrigation. Their study utilized indices such as SSI, STI, MP, and GMP to identify drought-tolerant genotypes, with Uzun emerging as the most resilient under water-limited conditions. These studies highlight the importance of drought tolerance indices in genotypes; however, many conventional indices primarily rely on absolute yield differences rather than accounting for relative yield performance within specific environmental contexts.

Although a plant’s genetic makeup remains constant across different environments, except for rare spontaneous mutations, genes alone do not fully determine its appearance or traits. Instead, genes allow for a range of expressions based on the plant’s genetic background, how it develops in specific tissues, and environmental factors (Rédei, 1998; Yan and Kang, 2002). The Additive Main Effects and Multiplicative Interaction (AMMI) and GGE biplots are some of the most commonly used methods by plant geneticists to evaluate genotype performance in diverse environments (Frutos et al., 2014). Sanadya et al. (2025) evaluated forage oat germplasm under varying growing conditions in conventionally and organically managed fields in Palampur, India, assessing GEI using GGE and AMMI biplot analyses. Over three cropping seasons (2019–2022), they studied 96 oat genotypes across inorganic and organic farming systems, observing significant GEI effects on green fodder yield and dry matter yield. The researchers identified highly stable genotypes for green fodder yield, stable genotypes for dry matter yield, and delineated mega-environments, each with specific genotypes exhibiting superior performance.

To address the limitations of existing drought selection methods, this study introduces the Drought Adaptation Index (DAI), a BLUP-derived index inspired by the Acid Soil Adaptation Index (ASAI) (Howeler et al., 1991). The DAI quantifies drought resilience by comparing biomass yield BLUP values under drought-stressed and well-watered conditions, enabling precise differentiation of very well-adapted, well-adapted, adapted, and unadapted genotypes. Unlike traditional indices that rely on absolute yield differences, DAI accounts for relative yield performance within a given environmental context. In addition to developing the DAI, this study calculated several established drought tolerance indices - including Stress Susceptibility Index (SSI), Stress Tolerance Index (STI), Geometric Mean Productivity (GMP), and Yield Stability Index (YSI) - to compare their effectiveness in identifying stress-resilient genotypes. By integrating multiple drought indices, this study provides a comprehensive assessment of drought tolerance and identifies the most reliable selection metrics for breeding programs. Specific objectives include evaluating the drought adaptation potential of a diverse set of switchgrass genotypes under contrasting water availability conditions, validating the DAI as a novel selection criterion, comparing DAI with other drought tolerance indices, analyzing genotype stability across years, and informing breeding decisions for sustainable biomass production. The findings are expected to provide valuable insights for improving biomass yield and drought resilience in switchgrass, with applications for both bioenergy production and forage systems.

## 2 Materials and methods

### 2.1 Experimental sites and plant materials

**FIGURE 1:**
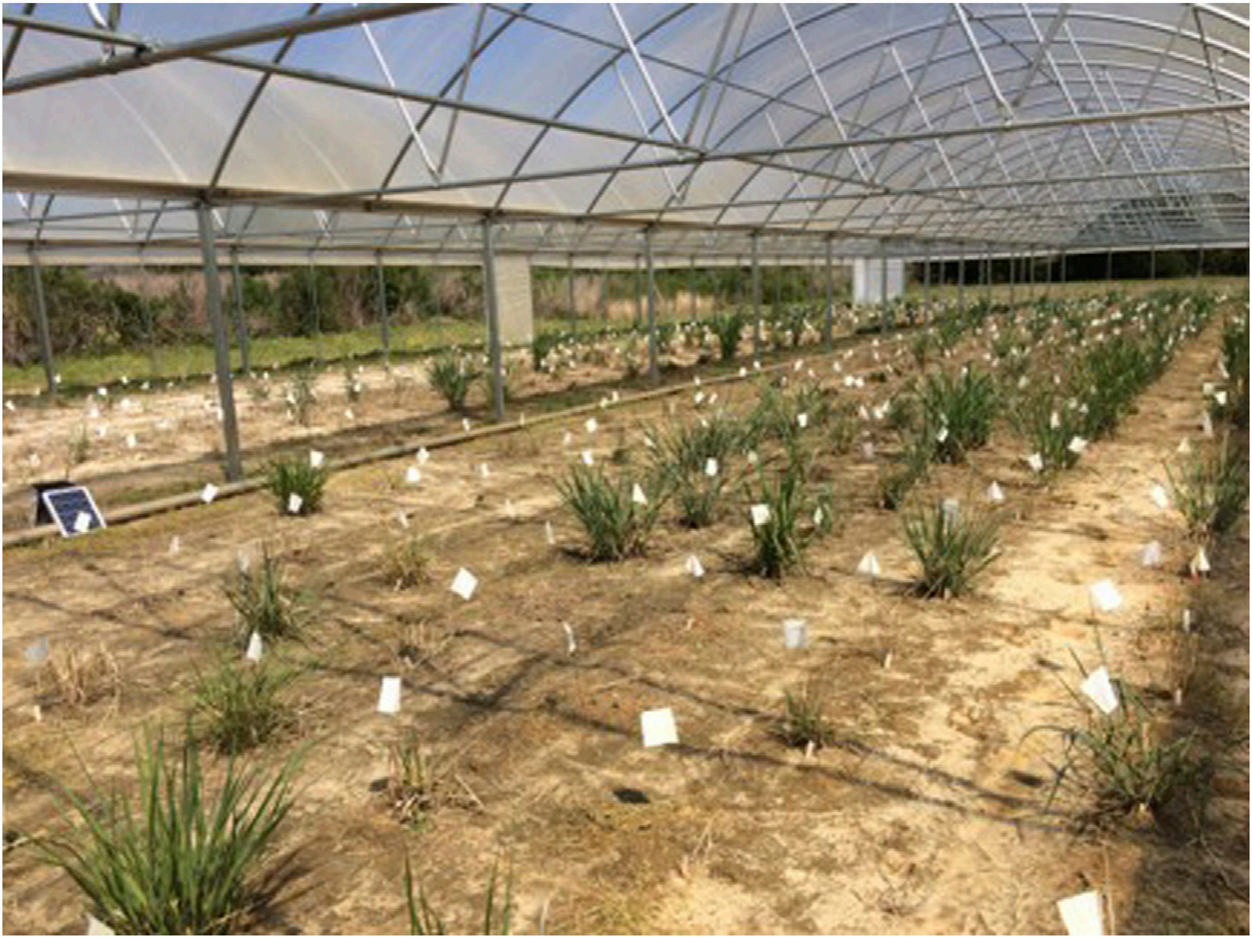
Switchgrass growing inside a rainout shelter, representing the water-stressed drought treatment or covered field (CV).

**FIGURE 2:**
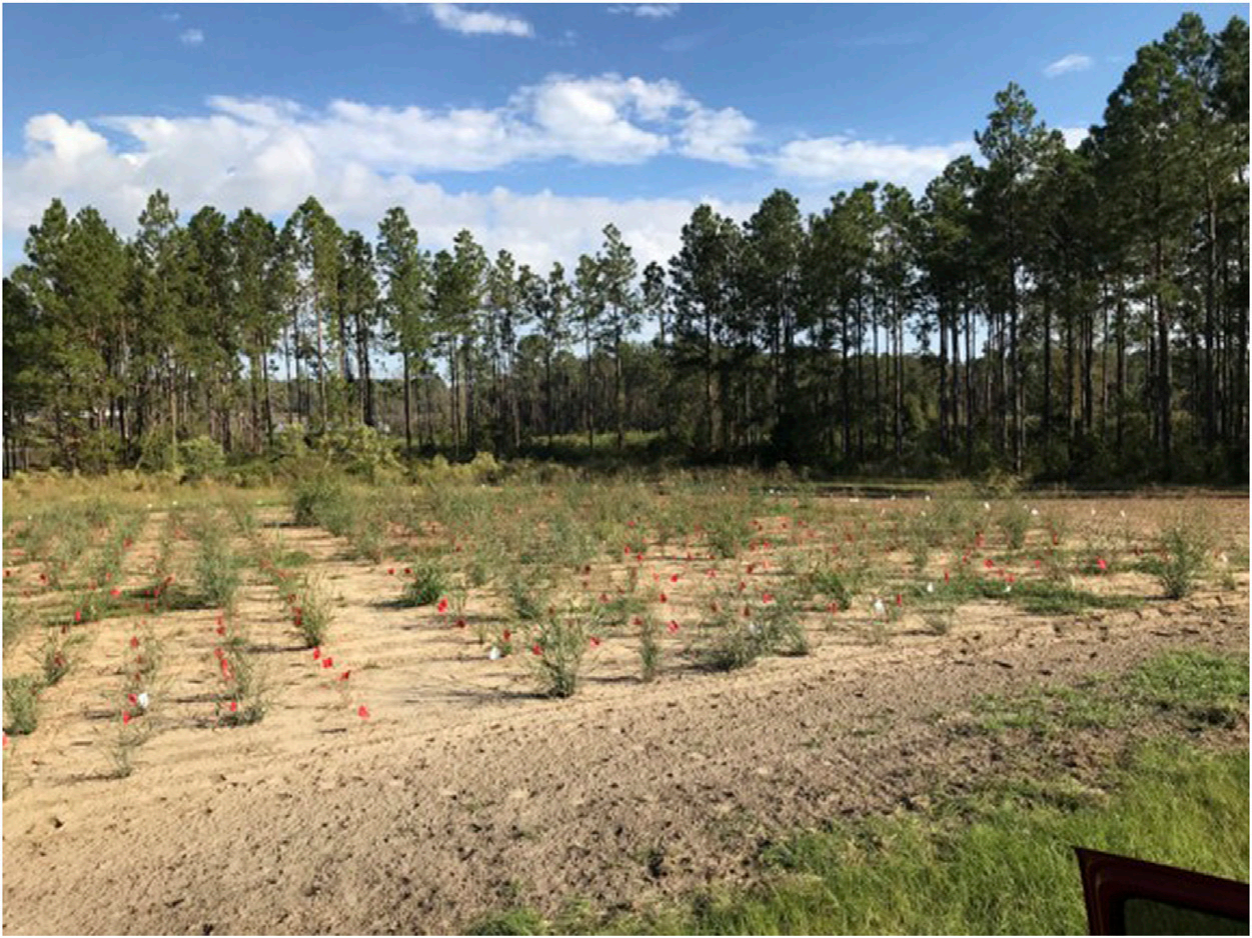
Switchgrass in a rainfed and irrigated control or uncovered treatment (UC).

The experimental sites were located at the University of Georgia (UGA) Gibbs Farm in Tifton, GA. The soil type is loamy sand. The experimental setup was a randomized complete block (RCB) design with three replications for each of the two treatments: drought-stressed (covered, CV; Figure 1) (31.441535° N, −83.580013° W, 116 m elevation) and well-watered control (uncovered, UC; Figure 2) (31.438345° N, −83.580185° W, 116 m elevation). Due to limited availability of tiller material during initial transplanting period, only two replications for each treatment were established in August 2018. The third replication for each treatment was propagated in the greenhouse and added in April 2019. Plants are spaced 0.9 m within each block (replication) in a grid layout of seven rows and 58 columns in the CV field and 14 rows and 29 columns in the UC field as shown in the spatial map (Supplementary Figure S1) as shown in the spatial map showing the spatial distribution of genotypes within each block, ensuring randomized planting and minimizing spatial biases. The x-axis denotes row positions, while the y-axis indicates column positions within each block. Each panel corresponds to one of the three replications (blocks), distinguished by separate facets. The genotypes are color-coded by replication: Blue (Block 1), Green (Block 2), and Red (Block 3). The presence of colored rectangles in each block represents plant genotypes, while white spaces indicate missing or unplanted areas. In plots where plants were missing or died, AP13 filler plants were used to minimize border and edge effects.

A total of 404 switchgrass genotypes were used in this study, representing different cytotypes and ecotypes. The distribution of these genotypes across different cytotypes and ecotypes is presented in Table 1 (Lovell et al., 2021). The dataset includes lowland, coastal, and upland classification, with a few unknown genotypes, primarily from tetraploid cytotypes and a few from octaploid cytotypes. Prior to the establishment of the experiment, the drought-stressed field was cropped with cotton and peanut from 2012 through 2017 and the control field was a long-term grassland consisting of bahiagrass (*Paspalum notatum*) and bermudagrass (*Cynodon dactylon*).

**TABLE 1:** The cytotype (octaploid, tetraploid, and unknown) and ecotype distributions among 404 switchgrass genotypes.

Ecotype	Octaploid	Tetraploid	Unknown	Total
Lowland	0	134	3	137
Coastal	0	112	10	122
Upland	0	98	1	99
Unknown n	9	18	19	46
Total	9	362	33	404

Source: Lovell et al. (2021).

### 2.2 Drought treatment, environmental control, and trial maintenance

To impose drought stress on the switchgrass genotypes, a rain exclusion shelter (55 m in length, 26 m in width, and 2.3 m in height, with a maximum height of 4.5 m at the peak of the hoop roof) was installed (Figure 1). The shelter is designed to block rainfall while maintaining adequate airflow. The side panels can be fully or partially opened or closed depending on weather conditions to balance ventilation and drought control. The shelter accommodated three blocks, each with a capacity of 406 plants. To monitor the water table, six piezometers (PVC well screen pipes) with a depth of 8 ft were installed at three key locations: one at the middle of the left border edge, one at the middle of the right border edge, one at the front between the first and second blocks, one at the front between the second and third blocks, one at the back between the first and second blocks, and one at the back between the second and third blocks. After the switchgrass establishment year, the drought plots were not irrigated beyond early spring fertilization, effectively creating a dry-down condition.

Weather conditions during the trial period (2019–2022) were obtained from the University of Geogia Weather Network (2025). These years showed substantial interannual and seasonal variability in both temperature and precipitation. Monthly average maximum temperatures (based on mean daily highs) ranged from 15.1 °C (January 2021) to 32.0 °C (May 2019), while monthly average minimum temperatures (based on mean daily lows) ranged from 5.1 °C (January 2019) to 19.5 °C (May 2019). Monthly total precipitation varied widely across years and seasons, from as low as 2.7 cm (May 2021) to as high as 22.3 cm (February 2021) (Supplementary Table S2). Although the drought fields were protected from rainfall using a rain-exclusion shelter and the control fields received scheduled irrigation, other weather factors such as air temperature and solar radiation were not controlled and fluctuated across years and seasons. These environmental variations may have influenced plant physiological responses and contributed to differences in genotype performance under both drought and control conditions.

A Bartlett Weather Alarm (Model RWL11X2, Bartlett Instrument Company, Fort Madison, IA, United States of America) was integrated into the system to monitor environmental conditions, including rainfall. The alarm detected precipitation events and assisted in operating the rainout shelter, ensuring that drought plots remained sheltered during rainfall. Additionally, a VentBoss 2.0 (Bartlett Instrument Company, Fort Madison, IA, United States of America) was installed to regulate airflow and mitigate excessive heat buildup. A Rain Bird irrigation system (Rain Bird Corporation, Azusa, CA, United States of America) was installed to enable precise and automated water application, while an AQUA TRAC system (AgSense, Huron, SD, United States of America) was used to monitor soil moisture levels and assist in managing irrigation efficiency.

**FIGURE 3:**
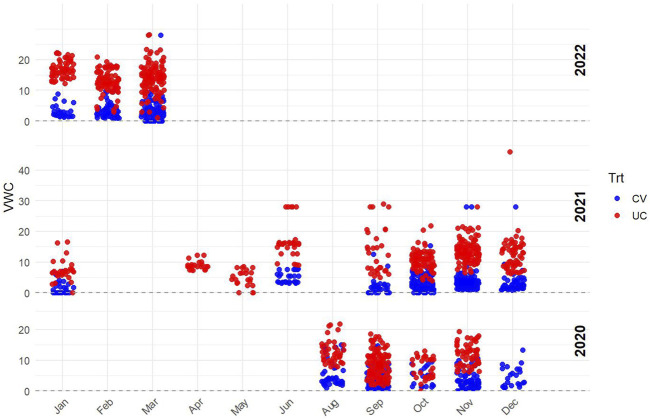
Volumetric water content (VWC) measured using a FieldScout TDR 350 Soil Moisture Meter (Spectrum Technologies, Inc., Aurora, IL) at a soil depth of 0–10 cm to assess moisture availability in the drought field (blue data points) and control field (red data points).

Soil moisture levels were monitored using a FieldScout TDR 350 Soil Moisture Meter (Spectrum Technologies, Inc., Aurora, IL), with volumetric water content (VWC) measured in both the CV and UC fields. Measurements were taken at multiple time points throughout the growing season at a soil depth of 0–10 cm to assess differences in soil moisture availability between treatments (Figure 3). Although this depth does not capture the entire rooting zone, it served as an indicator of surface soil moisture under drought.

A drip irrigation system was used to supply water to plants in both the drought and control sections. In the drought field, during the initial establishment year, drip irrigation was provided with one dripper per plant, installed using microtubing and polytube, with irrigation applied for 30 min per day. Following the establishment year, irrigation was withheld. In the control field, during the establishment year, irrigation was applied using a Hydrogol drip line with one dripper per plant position (both at the plant and between plants) for 15 min per day. In subsequent years, irrigation was provided as needed to maintain the moisture level at field capacity. An AQUA TRAC system (AgSense, Huron, SD, United States of America) was used as a regulator to monitor and manage irrigation.

Fertilizer was applied each spring through drip irrigation to both drought and covered fields using Jack’s Professional 20:20:20 water-soluble formulation. The application rate was 11.34 kg per treatment area (∼0.1018 ha), equivalent to approximately 111.35 kg/ha of total fertilizer. Given its balanced composition (20% each of nitrogen, phosphorus, and potassium), this corresponds to approximately 22.3 kg/ha of each nutrient (N, P, and K).

### 2.3 Biomass harvest

Biomass harvest was conducted for the first two replications during the fall of 2019, while subsequent harvests were carried out for all three replications each year from 2020 to 2022. In December, the plants were harvested at 10 cm height from ground. The plants at CV fields were harvested using chainsaw or hand sickles and the plant at UC field were harvested using Wintersteiger Cibus Harvester (Wintersteiger AG, Ried, Austria). To calculate the dry biomass yield of each switchgrass plant, a fresh biomass sample weighing between 50 g and 300 g was collected and dried in a forced-air dryer at a constant temperature of 55 °C for 5 days. The resulting dry matter percentage was then used to estimate the total dry biomass yield per plant.

### 2.4 Preliminary data processing

To ensure data reliability, we filtered the data by excluding yield values below 50 g plant^-1^ from further analyses. The raw dry biomass yield data were initially examined for distributional characteristics. Normality of the dataset was assessed using the Shapiro-Wilk test and visually confirmed with a Quantile-Quantile (QQ) plot. The initial raw and filtered distributions of dry biomass yields are illustrated in Supplementary Figures S2, S3, respectively. The filtered data was then used to identify outliers for removal using the Median Absolute Deviation (MAD) method, with the default MAD cutoff set to 6. The absolute MAD-scaled distance of each data point from the median was calculated as:

$$absMADAway=\frac{\left|data-median\left(data\right)\right|}{MAD\left(data\right)}$$

The value is considered an outlier if absMADAway > MADcutoff.

The spatial distribution of plant genotypes within each block was examined using a spatial map (Supplementary Figure S1) to confirm randomization and assess planting uniformity. The QQ plots (Supplementary Figure S4) were used to further examine skewness and tail behavior.

The Shapiro-Wilk test was conducted and a QQ plot was generated to assess whether the yield data follow a normal distribution for each year and treatment combination after curation and outlier removal. The Shapiro-Wilk test results, including the W statistic and p-values, are provided in Table 2. The QQ plots of dry biomass yield data after removal of outliers are presented in Supplementary Figure S4.

**TABLE 2:** The Shapiro-Wilk test to check distribution of the yield data (after curation and outlier removal) across different years and treatments.

Year	Treatment	Statistic (W)	*P* Value
2019	CV	0.81	0.00011
2019	UC	0.77	0.00002
2020	CV	0.80	0.00008
2020	UC	0.90	0.00730
2021	CV	0.79	0.00004
2021	UC	0.81	0.00008
2022	CV	0.84	0.00034
2022	UC	0.87	0.00184

### 2.5 Thin plate spline (TPS) correction

After outlier removal, a TPS regression model was applied to the yield data to account for spatial variation and ensure that interpolation and spatial corrections were not biased by extreme values. The TPS model was fitted using the Tps () function from the fields R package (Nychka et al., 2017), with spatial coordinates (row and column) as independent variables and yield values as the response variable. The residuals from the TPS model were extracted and added to the mean yield per plot to obtain TPS-adjusted values. These corrected values were then used for further statistical analyses.

BLUP values were estimated for each genotype using a linear mixed model (LMM) with genotype as a random effect. The model was fitted separately for each year and treatment using the lmer () function from the lme4 R package (Bates et al., 2015). Variance components were extracted from the model to compute repeatability ( R ) for dry biomass yield, calculated as:

$$R=\frac{V_{g}}{V_{p}}$$

where $V_{g}$ is the genetic variance and $V_{p}$ is the total phenotypic variance. V_g_ was extracted from the random effect variance component for genotype, and residual variance (V_e_) was obtained as the squared residual standard deviation from the linear mixed model. The estimated values of $V_{g}$ , $V_{p}$ , and repeatability for each year and treatment are presented in Table 3. The distribution of BLUP-predicted values was assessed using histograms to visualize variability across years and treatment conditions (Supplementary Figure S5). The ggplot2 package in R was used to generate histograms for BLUP distributions.

**TABLE 3:** Repeatability.

Year	Treatment	Genetic variance (V_g_)	Phenotypic variance (V_p_)	Repeatability (R)
2019	CV	530,654.89	839,764.08	0.63
2019	UC	353,617.41	728,616.16	0.49
2020	CV	331,640.61	997,327.24	0.33
2020	UC	378,310.46	1,021,137.62	0.37
2021	CV	207,718.63	668,504.68	0.31
2021	UC	792,607.53	1,654,216.53	0.48
2022	CV	62,063.54	271,506.21	0.23
2022	UC	413,679.04	916,472.04	0.45

### 2.6 Drought adaptation index (DAI)

We designed and calculated DAI for switchgrass genotypes following the procedure introduced by Howeler et al. (1991) for the Acid Soil Adaptation Index (ASAI). The DAI was calculated as follows:

$$Drought\;Adaptation\;Index\;\left(DAI\right)=\frac{Y_{CV}\times Y_{UC}\;}{{\bar{Y}}_{CV}\times {\bar{Y}}_{UC}}$$

where Y and $\bar{Y}$ represent individual yield BLUP performance (separately for each year) and the grand mean yield BLUP (for each year) for CV and UC treatments as indicated by the corresponding subscripts, respectively.

We also calculated nine other drought tolerance associated indices compiled and used by Sanchez-Reinoso et al. (2020) using BLUP predicted values for biomass yield under drought and control conditions to compare with the index DAI that we designed. While Sanchez-Reinoso et al. (2020) applied these indices to grain yield, we adapted them for biomass yield evaluation to assess drought tolerance in our study. The indices included: Stress Susceptibility Index (SSI), Tolerance (TOL), Mean Productivity (MP), Geometric Mean Productivity (GMP), Stress Tolerance Index (STI), Yield Stability Index (YSI), Yield Index (YI), Harmonic Mean (HM), and Drought Sensitivity Index (DSI). These indices along with DAI were computed using custom functions in R and applied to the dataset using the dplyr package. These nine indices were computed based on yield under stress (Y_s_), non-stress (Y_p_), the mean yield under stress (Ȳ_s_), and non-stress (Ȳ_p_) conditions following the methodology described by Sanchez-Reinoso et al. (2020). The index formulae are defined as follows:

$$SSI=\frac{1-\left[Y_{s}{/Y}_{p}\right]}{1-\left[{Ȳ}_{s}{/Ȳ}_{p}\right]}$$

$$TOL=Y_{p}-Y_{s}$$

$$MP=\frac{Y_{p}+Y_{s}}{2}$$

$$GMP=\sqrt{Y_{p}\times Y_{s}}$$

$$STI=\frac{Y_{s}{\times Y}_{p}}{{\left({Ȳ}_{p}\right)}^{2}}$$

$$YSI=Y_{s}/Y_{p}$$

$$YI=Y_{s}{/Ȳ}_{p}$$

$$HM=\frac{2Y_{s}Y_{p}}{Y_{s}+Y_{p}}$$

$$DSI=\frac{Y_{s}{-Y}_{p}}{Y_{p}}$$

**FIGURE 4:**
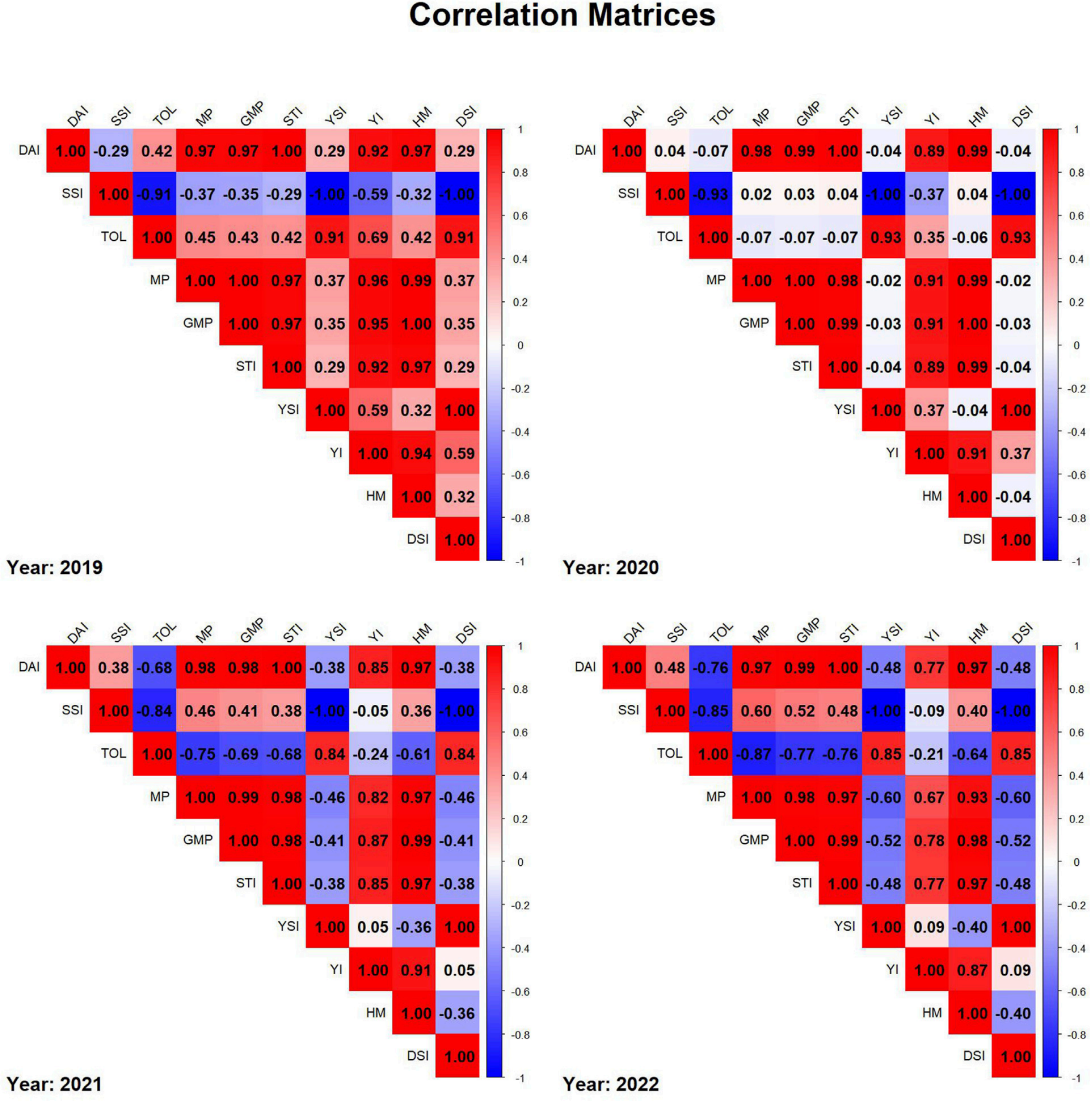
Correlation matrices of drought indices for each year from 2019 to 2022, where red represents positive correlations, blue represents negative correlations, and color intensity reflects the strength of the correlation.

**FIGURE 5:**
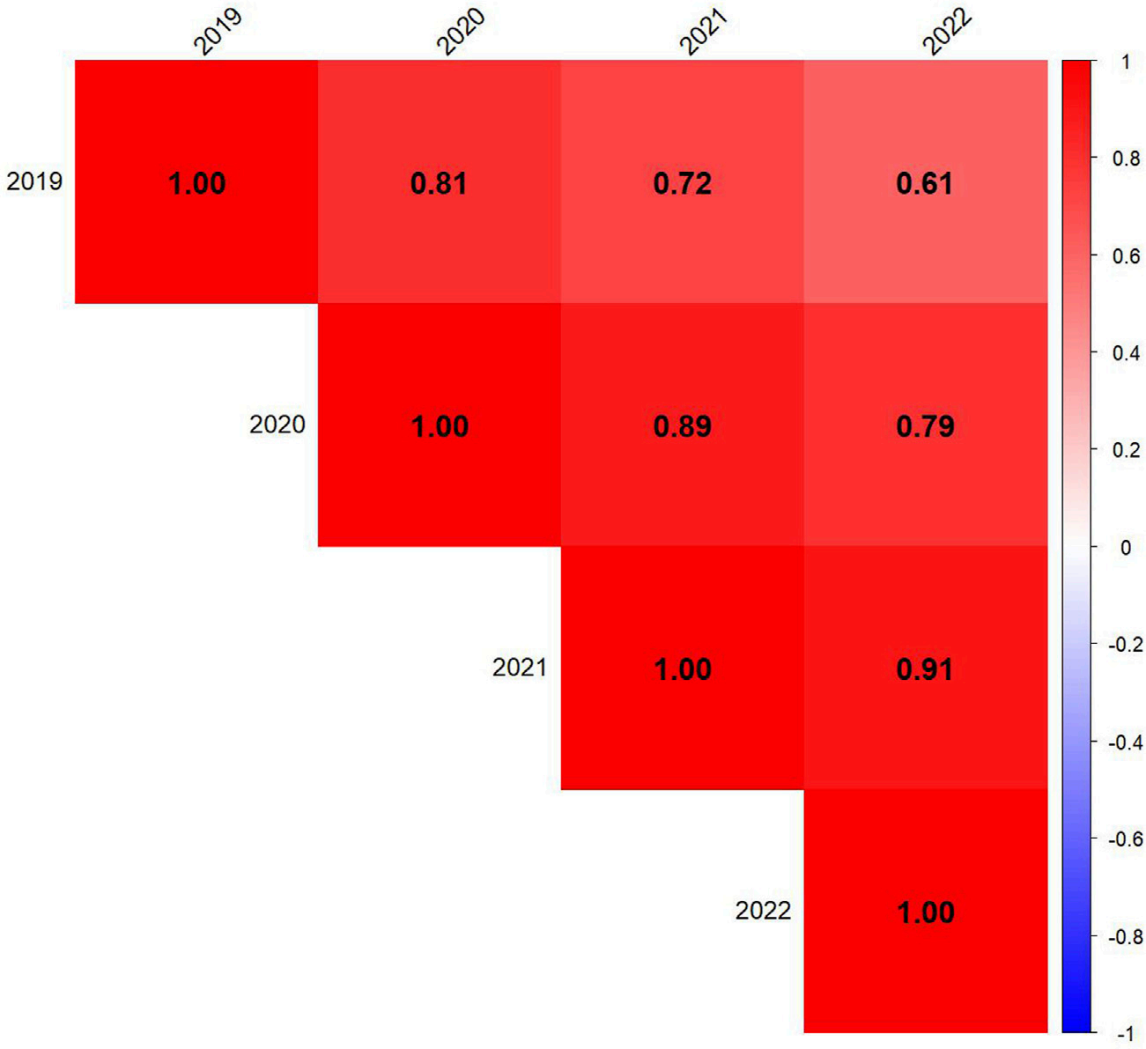
Correlation among 4 years to assess the stability of the Drought Adaptation Index (DAI). Red represents positive correlations, blue represents negative correlations, and color intensity reflects the strength of the correlation.

To evaluate drought tolerance and compare indices, Pearson correlation matrices were constructed for each year (2019–2022). Data for each year was extracted separately, removing non-numeric columns such as genotype identifiers to ensure accuracy. The correlation matrices were computed using complete observations and visualized with the corrplot package in R. The correlation plots were arranged in a 2 × 2 layout, with each subplot representing a different year. The corrplot function was used with a blue-to-red color gradient, and the final heatmaps were generated using R packages corrplot and dplyr (Figure 4). To assess the temporal stability of DAI across years, Pearson correlation coefficients were computed among DAI values across years using cor () function in R (Figure 5).

### 2.7 Visualization of DAI isoline curves

To visualize dry biomass yield stability across drought and control conditions, DAI isoline curves were generated separately for each of the 4 years (2019–2022). Data was first filtered to retain only numeric yield BLUP values for CV and UC treatments, removing any missing entries. For each year, genotype yield values were plotted on a scatter plots, with CV yield on the x-axis and UC yield on the y-axis. A custom function (C1_Fn) was used to compute the DAI threshold constants (C1 values) based on mean and standard deviations of yields under both conditions. Three C1 values corresponding to different standard deviation levels 0, 1, and 2 (SD0, SD1, SD2) were calculated to define isoline reference curves:

Blue (C1_SD_0_): Baseline stability threshold.

Red (C1_SD_1_): Moderate stability threshold.

Orange (C1_SD_2_): Higher stability threshold.

**FIGURE 6:**
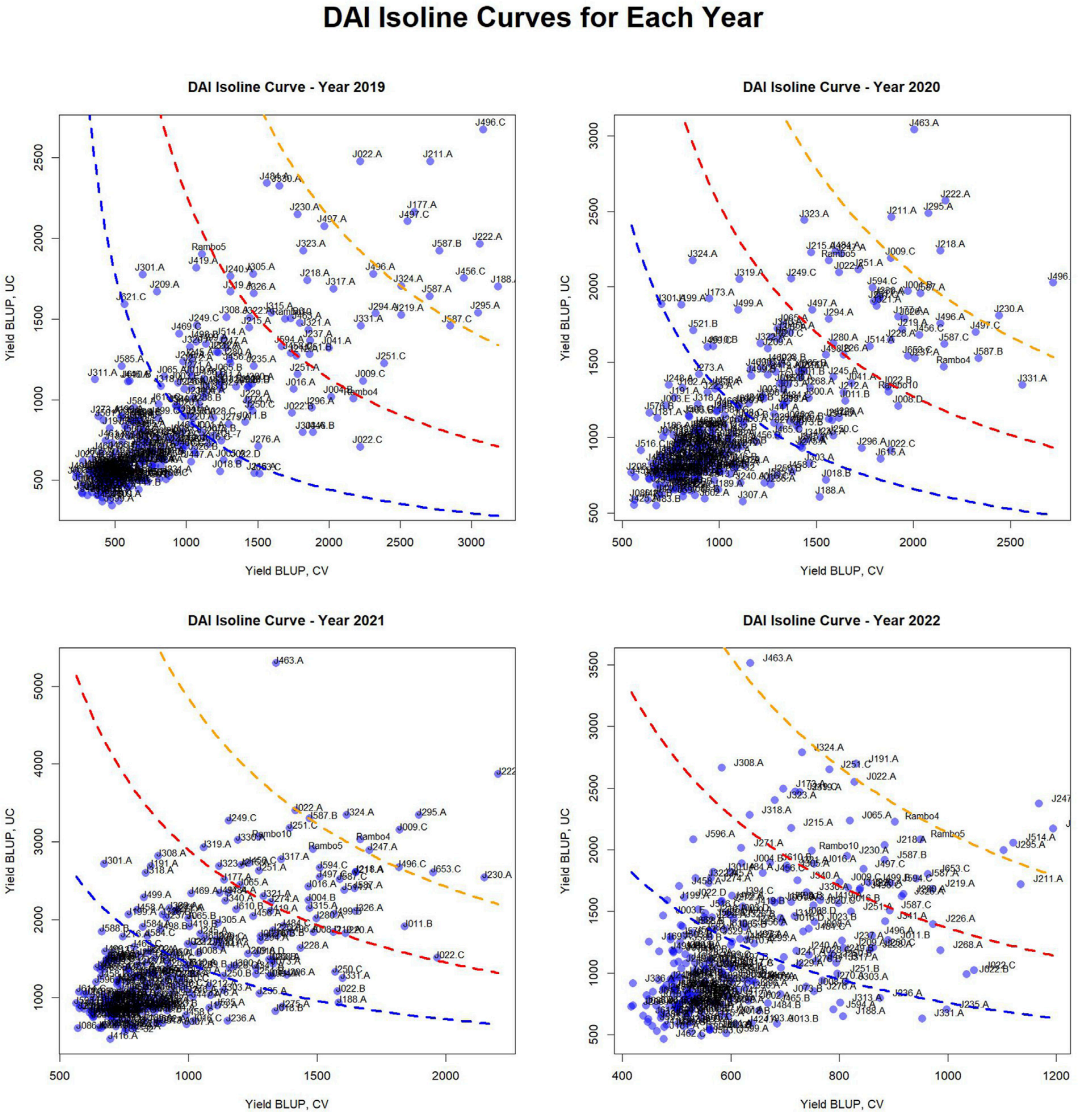
Drought Adaptation Index (DAI) isoline curves and classification of genotypes. These plots depict the distribution of genotypes based on their yield BLUP values in CV and UC conditions, with overlaid isoline curves representing different C1 constants, i.e., drought adaptation index threshold values (based on standard deviation values: 0, 1, and 2), which indicate varying degrees of drought stress impact. The plots categorize genotypes into four adaptation groups: very well-adapted (above the orange isoline), well-adapted (above the red isoline), adapted (above the blue isoline), and unadapted (below the blue isoline) for each year.

These isoline curves were overlaid onto the scatter plots to depict varying degrees of drought stress impact. Table 4 presents the C1 constants for each year. Genotype classification based on their positioning relative to the isolines is presented in Figure 6 and Supplementary Table S1. The plots were arranged in a 2 × 2 layout, representing individual years. This approach provides a graphical representation of genotype performance, aiding in selection strategies for drought resilience breeding.

**TABLE 4:** Drought adaptation index constants based on mean, mean + 1 SD, and mean + 2 SD.

Year	C1_SD_0_	C1_SD_1_	C1_SD_2_
2019	1	2.6	4.8
2020	1	1.9	3.2
2021	1	2.0	3.4
2022	1	1.8	2.8

### 2.8 GGE and AMMI biplots

A data-subset consisting of 24 genotypes and 8 environments was used for the analysis of GGE and AMMI biplot models. The environments were formed by combining two letter labels for treatments with the last two digits of the year. CV and UC represent drought and control treatments, respectively, while 19, 20, 21, and 22 correspond to the years 2019 through 2022. The dataset included BLUP-predicted biomass yield values across four drought environments (CV19, CV20, CV21, CV22) and four control environments (UC19, UC20, UC21, UC22). Missing values were imputed using the lowest observed value in each respective column to maintain data consistency. Only one missing value in UC19 for genotype J191. A was replaced with 539.2762, the minimum recorded value in that column. The biplot data analyses were performed using Genstat 24 (VSN International, 2024).

## 3 Results

### 3.1 Yield data distribution and normality assessment

The Shapiro-Wilk test results, summarized in Table 2, provided statistical evidence of deviation from normality (p < 0.05 for all cases). The W statistics ranged from 0.77 to 0.90, indicating varying degrees of skewness. The distributions exhibited a right-tailed skewness, with most observations concentrated at lower values, as shown in Supplementary Figure S2. After filtering out data points below 50 g plant-1, the distribution remained skewed but with a reduced range, as seen in Supplementary Figure S3. All QQ plots showed substantial deviations from the 45-degree reference line, particularly in the tails, indicating non-normal yield data (Supplementary Figure S4) consistent with the Shapiro-Wilk test results.

### 3.2 Soil moisture measurement using TDR 350

The recorded VWC values confirmed a consistent moisture deficit in CV, validating the effectiveness of the imposed drought conditions. The data show a clear separation between the two treatments, with CV consistently exhibiting lower VWC values compared to UC. This confirms that the drought-imposed conditions effectively reduced soil moisture availability in the CV field. Variability in the UC treatment likely resulted from natural fluctuations due to rainfall and soil characteristics.

### 3.3 BLUP distribution across years and treatments

The distributions of BLUP-predicted values across years and treatments (Supplementary Figure S5) highlighted variations in spread and treatment effects. The histograms revealed a right-skewed distribution, with a majority of genotypes showing lower BLUP values and fewer genotypes exhibiting exceptionally high values. The spread of values differed slightly across years, with some years displaying a broader range of predicted values. Additionally, differences in distribution patterns were observed between CV and UC treatments, suggesting a potential treatment effect on yield performance.

### 3.4 Repeatability (R)

Repeatability (R) values (Table 3) for dry biomass yield ranged from 0.23 to 0.63 across years and treatments. The highest repeatability was observed in the drought treatment in 2019 (0.63), indicating greater consistency in genotype performance under drought stress in that specific year. However, repeatability values were generally lower in drought-treated fields compared to control, highlighting the increased environmental influence and variability under drought stress.

### 3.5 Drought indices and correlation matrices

In 2019 correlation matrix, strong positive correlations were observed among MP, GMP, STI, and YSI, indicating their interdependence in evaluating drought tolerance. SSI showed a strong negative correlation with DAI and other yield-related indices, suggesting that higher stress susceptibility is associated with lower stability in drought conditions. The correlation matrix in 2020 is similar to 2019 as MP, GMP, and STI were positively correlated. However, the correlation of TOL with DAI, MP, GMP, STI and HM was weaker. The correlation matrix in 2021 showed correlation between SSI and stability indices (MP, GMP, STI) towards moderate positive and a high negative correlation between SSI with TOL. TOL exhibited negative correlations with yield stability, indicating its limited predictive ability for drought tolerance in 2021. In 2022, a strong correlation between DAI, MP, GMP, and STI was again observed, reinforcing their reliability in drought assessment. SSI showed an inverse relationship with drought resilience indices in 2022. Overall, the matrices show consistent patterns across years, helping in the selection process for drought tolerance assessment.

### 3.6 Stability of DAI across years

The correlation matrix shows strong positive correlations between yearly DAI values, indicating consistency in genotype responses to drought stress over time (Figure 5). The highest correlations were observed between consecutive years, such as 2020–2021 (r = 0.89) and 2021–2022 (r = 0.91), suggesting that genotypic drought susceptibility rankings remained relatively stable in sequential growing seasons. The correlation between 2019 and later years gradually decreased (2019–2022: r = 0.61), indicating potential environmental variability or shifts in drought response dynamics over time.

### 3.7 DAI isoline curves and genotypic classification

The scatter plots reveal a concentration of genotypes with lower yield BLUP values, while some genotypes with higher yield performance are spread across the plots. The curved isolines, color-coded as blue (SD0), red (SD1), and orange (SD2), provide a reference for identifying genotypes with higher yield stability across conditions. The positioning of genotypes relative to these isolines highlights differences in drought resilience across years.

The classification of genotypes based on their adaptation across different years is summarized in Figure 6, Supplementary Table S1. The table categorizes genotypes into four adaptation groups—very well-adapted, well-adapted, adapted, and unadapted—for each year. This classification highlights variations in genotype performance over time, offering insights into adaptation trends and resilience.

Notably, several genotypes were consistently classified as very well-adapted across multiple years, demonstrating strong adaptability and stability under varying conditions. These include J222.A and J295.A, which remained very well-adapted for four consecutive years, while J496.C and J463.A maintained this status across 3 years. Additionally, J211.A, J324.A, J587.B, J230.A, and J247.A were classified as very well-adapted for 2 years, indicating their potential for stable performance in diverse environments.

Conversely, some genotypes were classified as very well-adapted only in specific years, suggesting potential environmental influences or GEI. In 2019, J022.A, J177.A, J188.A, J456.C, J497.C, and J587.A were uniquely identified as very well-adapted. In 2020, J218.A was classified in this category, whereas in 2021, J009.C, J653.C, and Rambo4 were uniquely recognized as very well-adapted. In 2022, J191.A and J514.A were the only genotypes categorized as very well-adapted. This analysis underscores the importance of identifying stable, high-performing genotypes for long-term breeding and genetic improvement programs. The consistent classification of certain genotypes across multiple years suggests their robustness, while the year-specific classification of others highlights potential interactions with environmental factors.

### 3.8 Principal components and environmental relations

**FIGURE 7:**
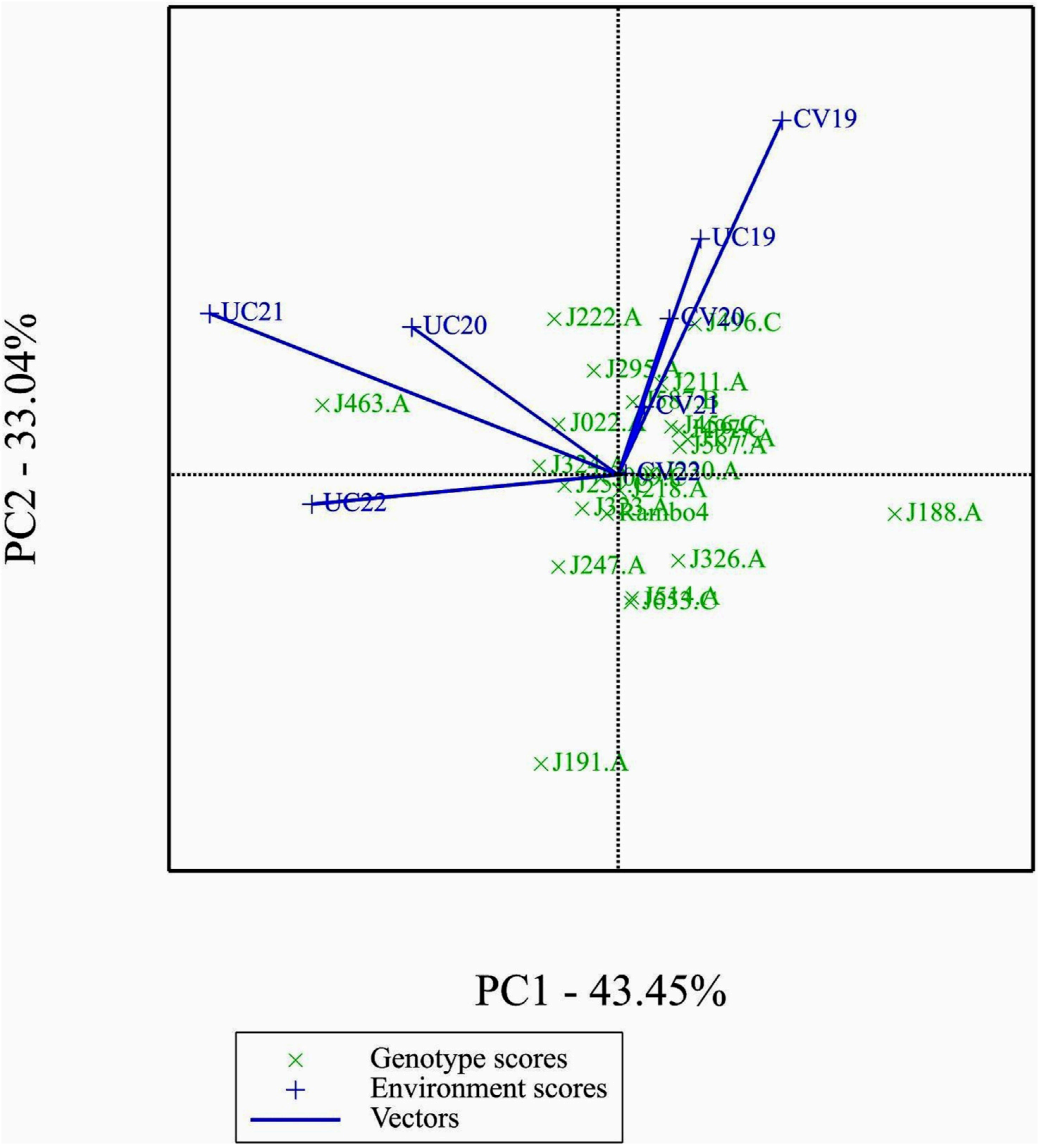
GGE biplot.

The GGE biplot (Figure 7), which integrates both genotype main effects (G) and GEI into a single analysis using PC1 and PC2, explained 76.49% of the total variation. The biplot provided a clear separation between environments based on treatment conditions, distinguishing drought and control treatments. Notably, most environments clustered by treatment, except for UC19, which was positioned closer to the drought environments. This spatial proximity in the biplot may be attributed to the reduced environmental contrast between treatments in 2019.

### 3.9 Which-won-where biplot analysis

**FIGURE 8:**
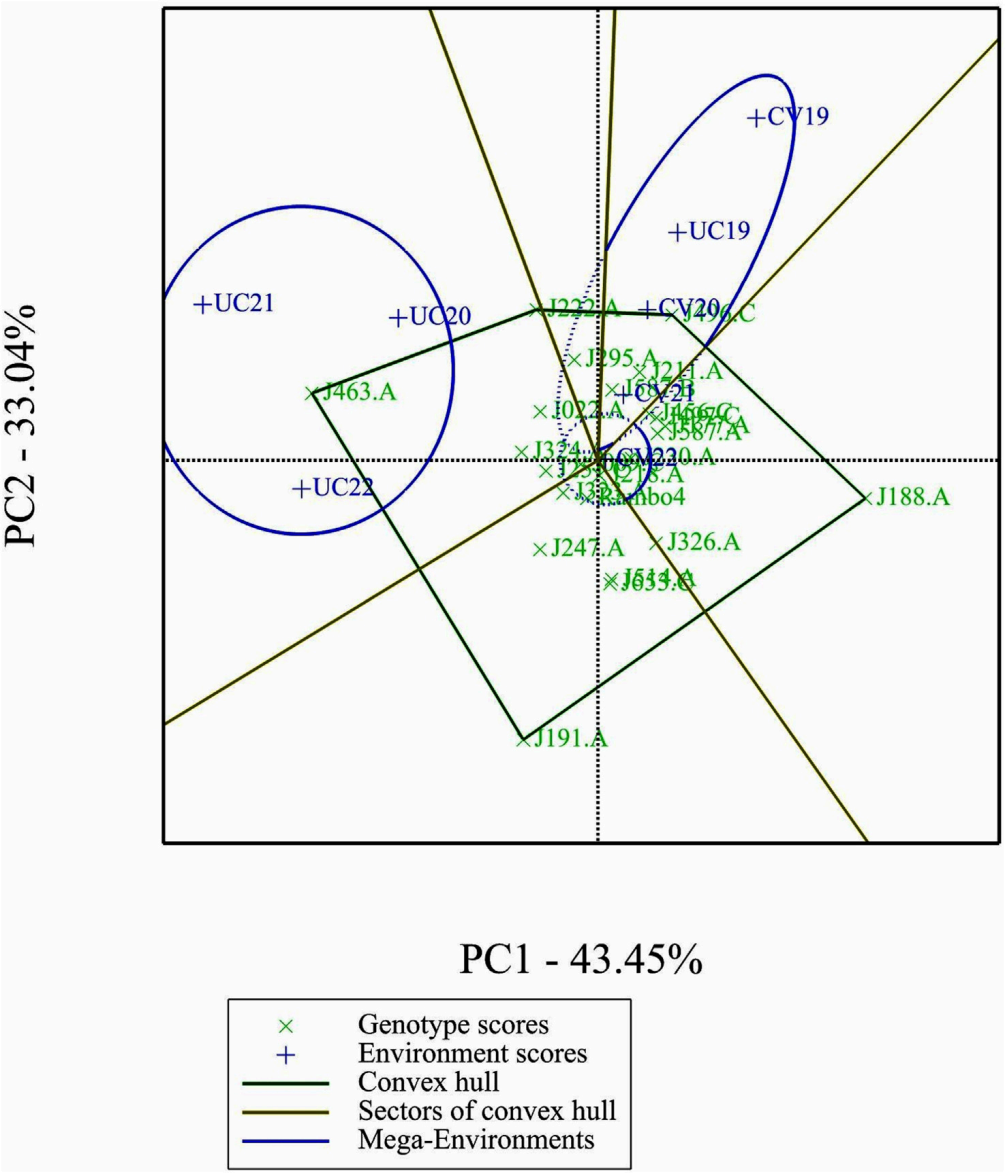
‘Which-Won-Where’ analysis.

The which-won-where biplot identified two mega-environments, each favoring specific genotypes (Figure 8). J463.A emerged as the most adapted genotypes in the mega environment formed by UC20, UC21, and UC22 as indicated by the position of the genotype in the vertex of the polygon. Genotype J222.A being in the same mega-environment as J463, they share similar adaptation patterns. Genotype J496.C is a winner in the mega-environment formed by the environments CV19, CV20, and UC19.

### 3.10 Mean performance and stability of genotypes

**FIGURE 9:**
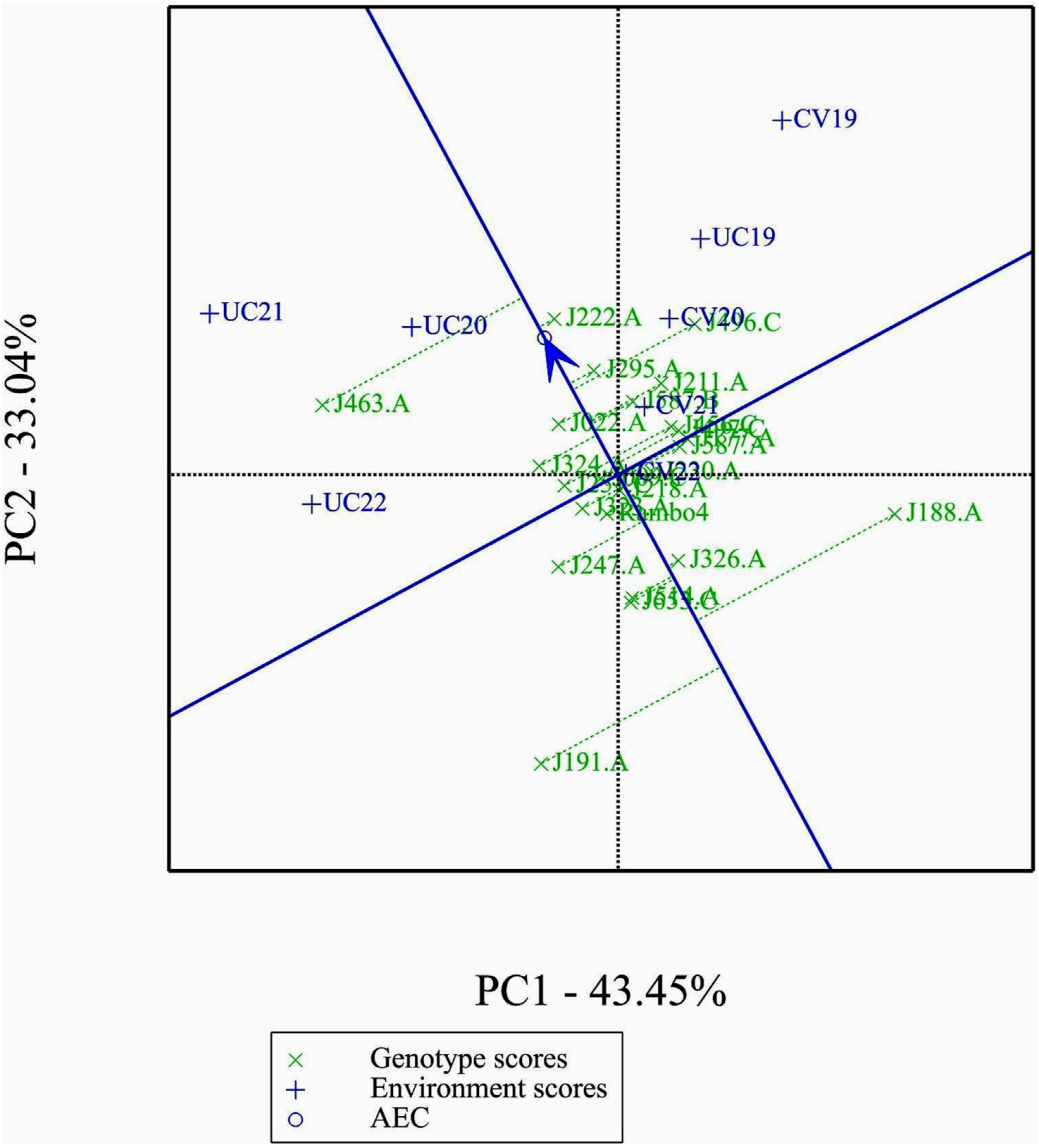
Mean performance and stability of genotypes.

Figure 9 shows clear differences in mean performance and stability among the genotypes. The single-arrowed Average Environment Coordination (AEC) abscissa points toward higher mean performance, whereas genotypes positioned in the opposite direction have lower yield potential. The variability of a genotype is indicated by its position along the AEC ordinate, which passes through the biplot origin and is perpendicular to the AEC abscissa. Stability is determined by proximity to the AEC abscissa, with more stable genotypes positioned closer to it. Among the genotypes, J463.A had the highest mean yield performance, followed by J222.A, J295.A, and J496.C. Despite its high yield potential, J463.A also exhibited greater variability. Therefore, genotypes such as J222.A and J295.A, which combined good stability with high yield potential, are promising candidates for selection. J191.A and J188.A were the least stable and lowest-yielding genotypes, indicating poor adaptability across environments.

### 3.11 Ranking genotypes relative to the ideal genotype

**FIGURE 10:**
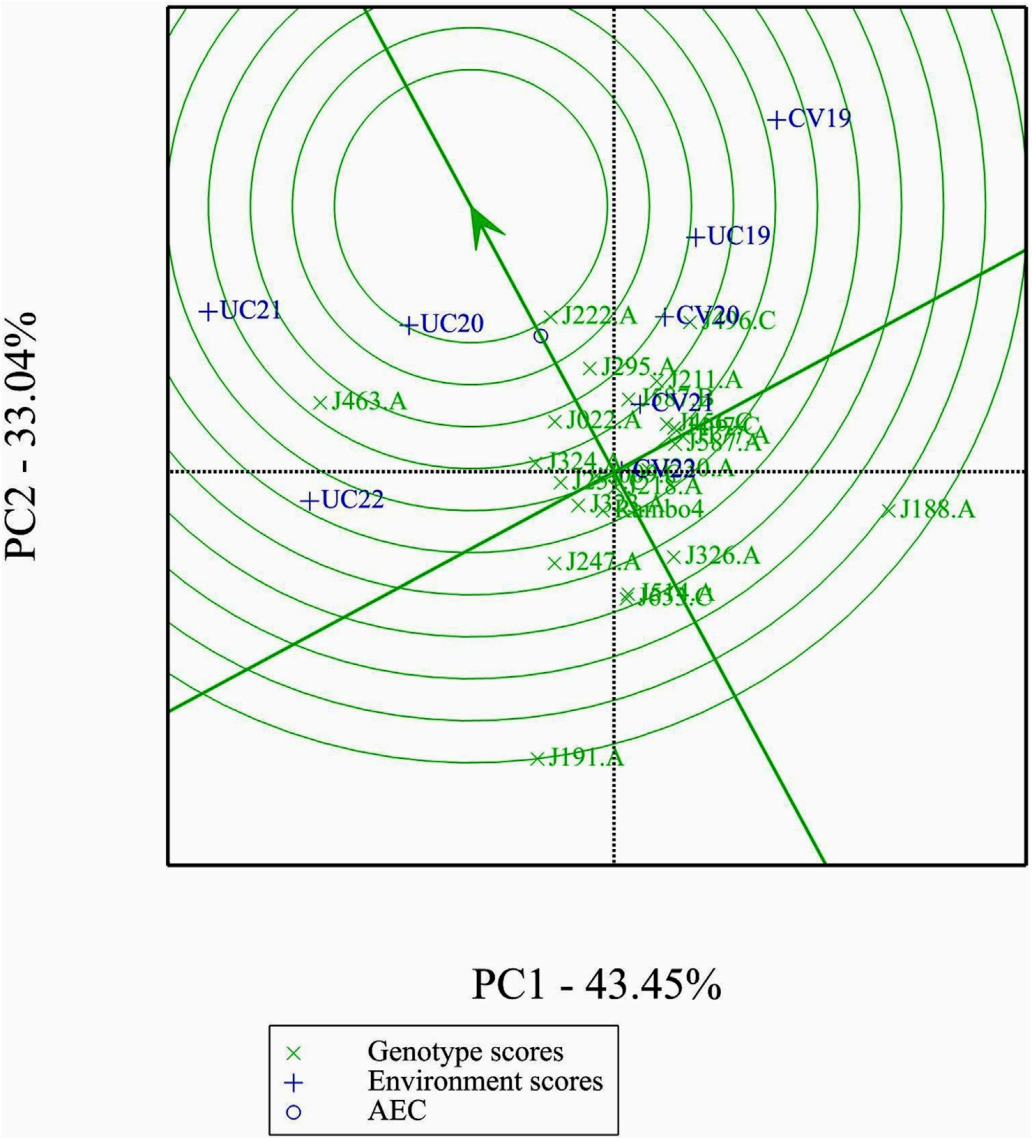
Performance of genotypes with respect to the ideal genotype.

The GGE biplot ranking analysis (Figure 10) provides a visual assessment of the mean performance and stability of genotypes relative to an ideal genotype. The ideal genotype, represented by the center of the concentric circles, serves as a reference for identifying the most desirable accessions. Genotypes closer to this point exhibit both high yield and stability, while those farther away show either lower performance or greater variability. Among the 24 genotypes, J222.A is closest to the ideal genotype, indicating its superiority in both yield potential and stability across environments. Other promising genotypes include J295.A and J496.C, which show a favorable balance of performance and stability. In contrast, J191.A and J188.A are positioned farther from the ideal point, suggesting greater variability and lower desirability for selection.

### 3.12 Comparison of specific genotypes and environments

**FIGURE 11:**
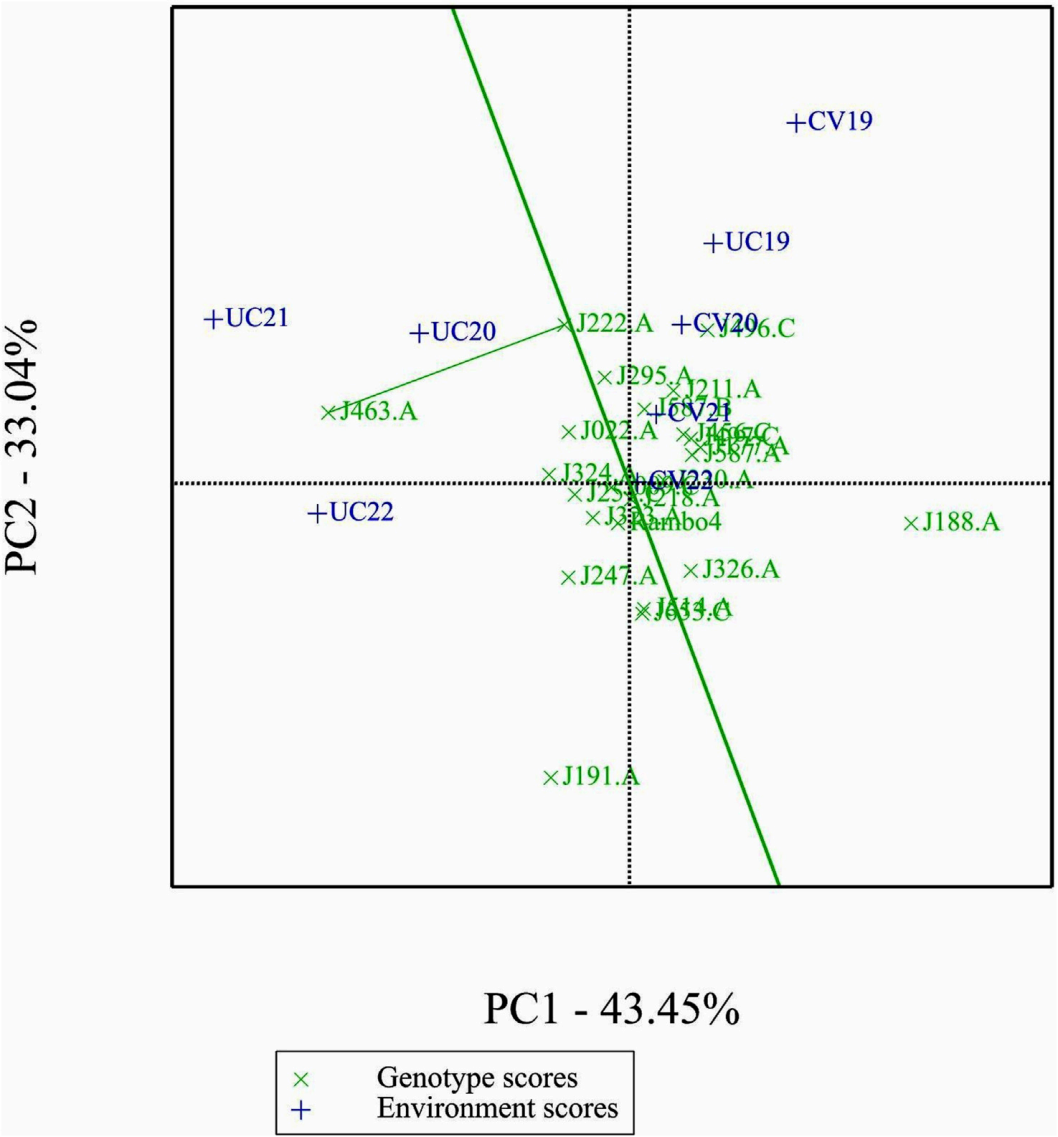
Comparison of genotypes J222.A, and J463.A.

**FIGURE 12:**
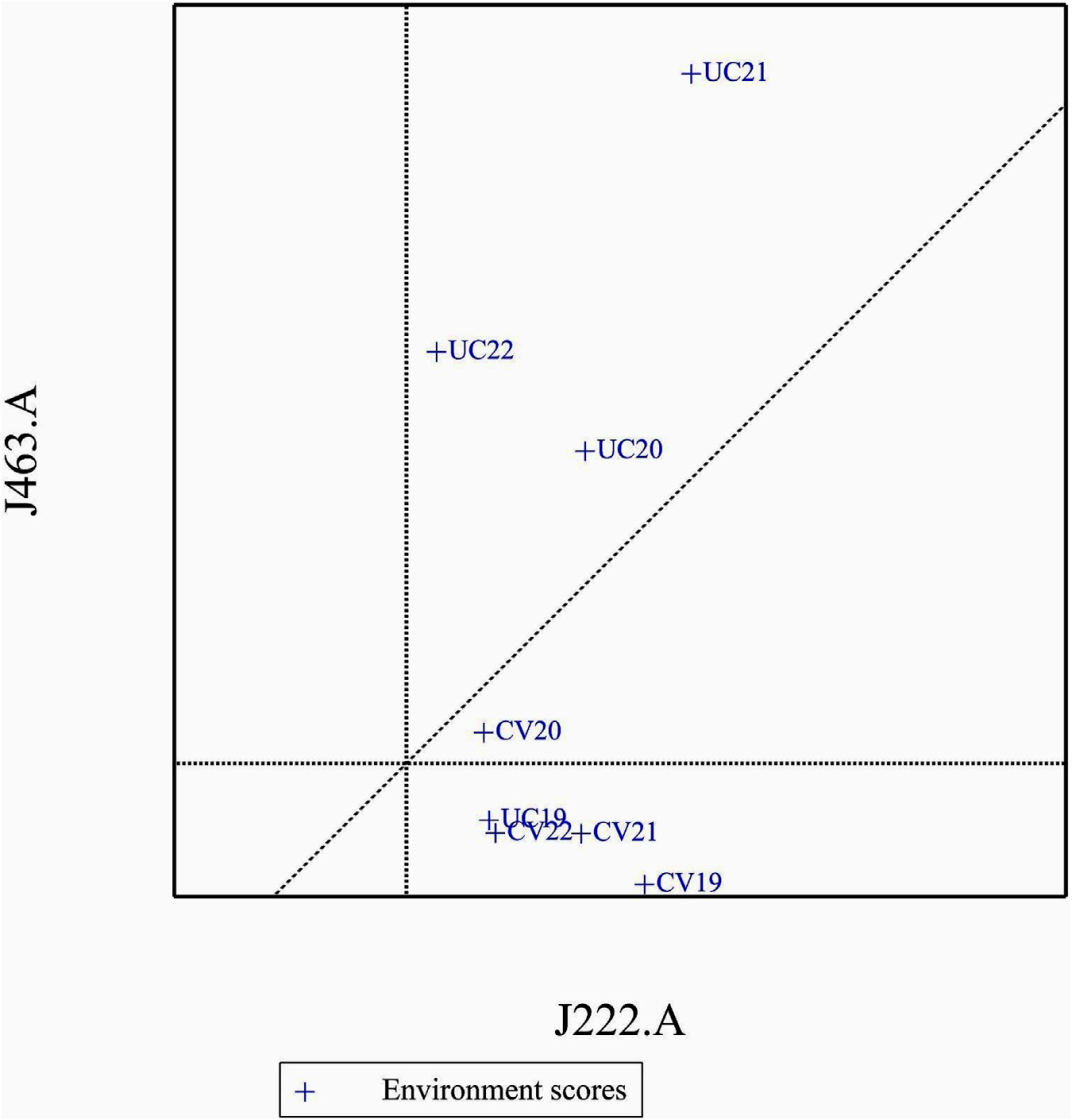
Comparison of genotypes J222.A and J463.A, based on environments.

**FIGURE 13:**
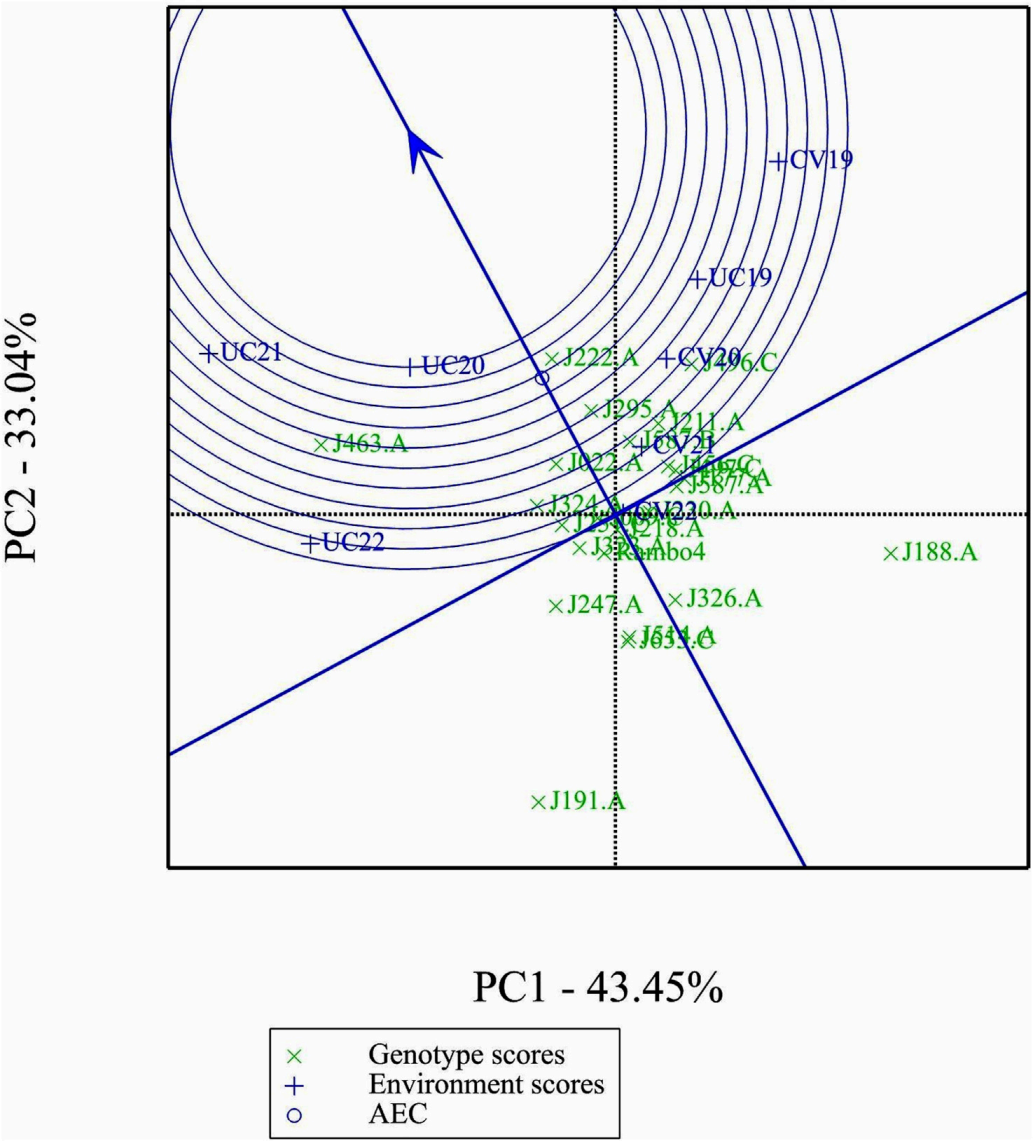
Ranking environments based on discriminating ability and representativeness for yield data.

In comparison of two specific genotypes, both J222.A and J463.A are high yielding but J222.A is more stable compared to J463.A (Figure 11). In terms of performance of genotypes in the environment, in general J222.A can be recommended when environment is drought prone, whereas J463.A can be recommended in stress-free normal environments (Figure 12). GGE biplot suggests UC20 and UC21 as better environments in terms of discriminating ability and representativeness (Figure 13).

### 3.13 Mean performance and interaction effects based on AMMI and ANOVA

**FIGURE 14:**
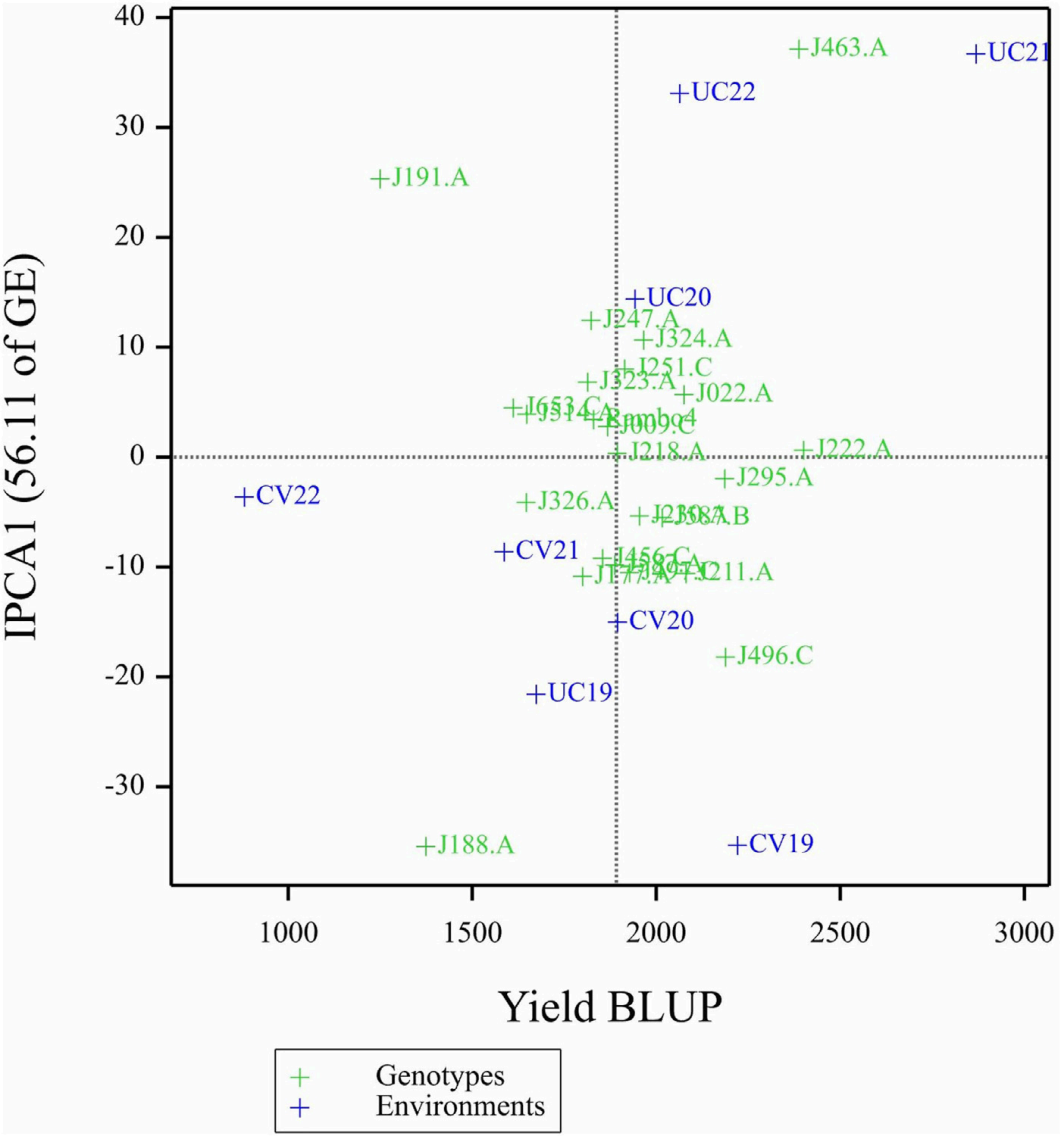
Biplot of genotype mean performance (yield as BLUP-predicted value) versus first interaction principal component (IPCA1) scores.

**FIGURE 15:**
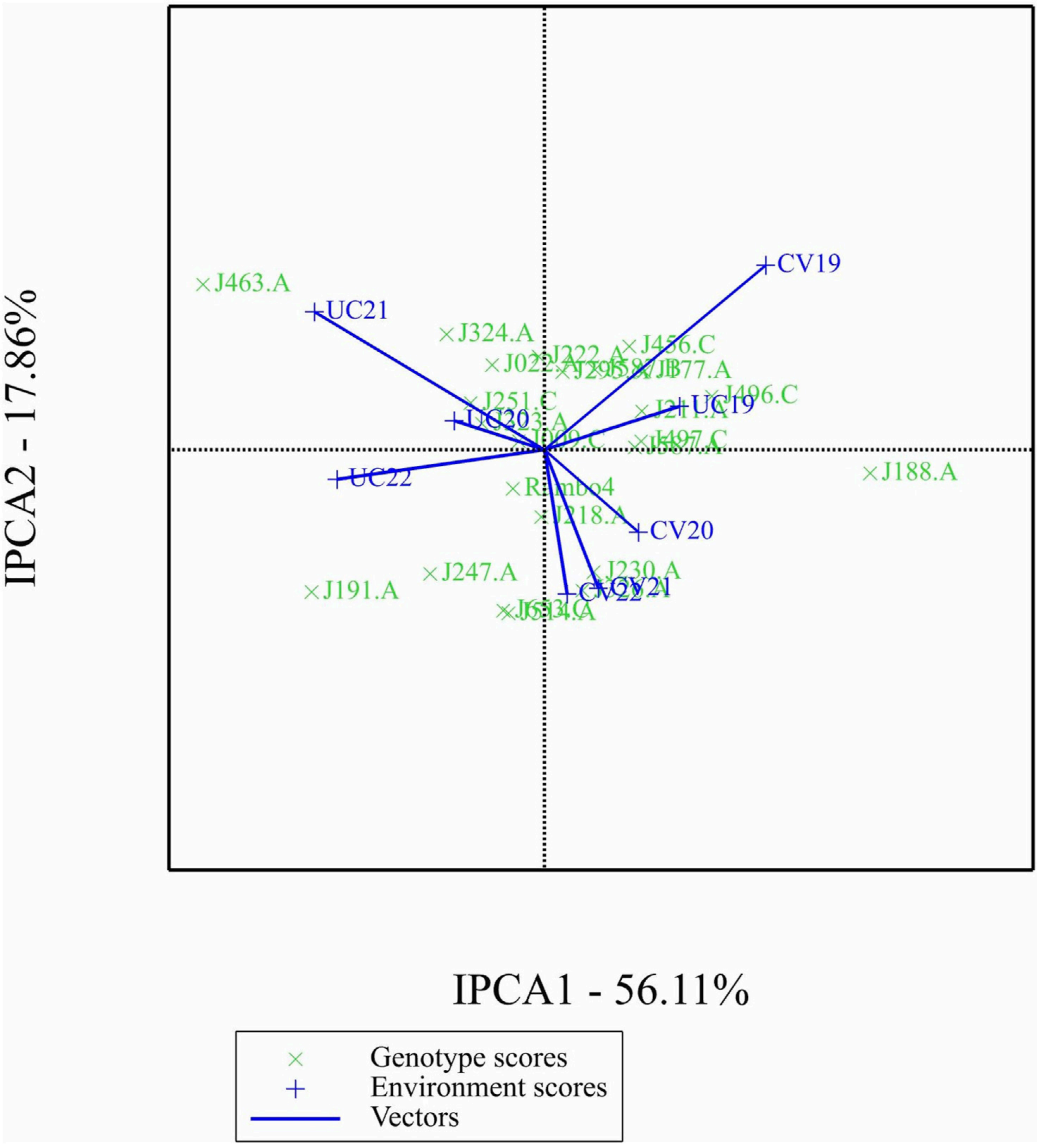
Biplot of first interaction principal component (IPCA1) scores versus second interaction principal component (IPCA2) scores.

The AMMI analysis showed significant effects of genotype (G), environment (E), and their GEI on biomass yield (p < 0.001; Table 5). Among these, the environment contributed the most to the total variation, followed by GEI. The interaction was further partitioned using interaction principal component axes, with the first two components explaining 56.11% (IPCA1) and 17.86% (IPCA2) of the interaction sum of squares, respectively. Mean performance and IPCA scores for each genotype and environment are presented in Table 6. Genotypes with higher mean yield and lower absolute IPCA scores were considered stable and broadly adapted. For example, J222.A and J295.A had relatively high yields and IPCA1 values near zero (Figure 14), indicating high and stable performance. In contrast, J463.A exhibited the highest mean yield but also high positive IPCA1 (37.13) and IPCA2 (17.98) scores (Figure 15; Table 6; Supplementary Figure S6), suggesting strong interaction with specific environments and lower stability. Among environments, UC21 had the highest mean yield (2,869 g plant^-1^), followed by CV19 (2,221 g plant^-1^), while CV22 was the least productive (881 g plant^-1^) (Table 6). CV19 (IPCA1: 35.33, IPCA2: 29.45) exhibited a contrasting interaction pattern and CV22 had negative values for both IPCA1 and IPCA2, indicating poor adaptability and low yield potential. Three AMMI biplots illustrate different aspects of GEI. The genotype mean performance versus IPCA1 biplot (Figure 14) highlights genotypes combining high yield and stability - those near the origin on the y-axis are considered stable. The IPCA1 versus IPCA2 biplot (Figure 15) visualizes crossover interactions, where proximity between genotype and environment points suggests positive interaction, and positioning in opposite quadrants indicates specific adaptation. The genotype mean performance versus IPCA2 biplot (Supplementary Figure S6) captures residual interaction effects not explained by IPCA1 and helps identify genotypes with high yield and low variability in IPCA2. Together, the AMMI ANOVA (Table 5), mean and interaction scores (Table 6), and the three biplots (Figures 14, 15) support the identification of stable, high-performing genotypes such as J222.A and J295.A, as well as specifically adapted genotypes like J463.A. Environments such as UC21 and CV19 demonstrated strong discriminatory ability and interaction patterns, making them particularly informative for genotype evaluation and selection.

**TABLE 5:** Analysis of variance (ANOVA) from AMMI model for genotype-by-environment interaction (GEI) of biomass yield on BLUP-predicted values.

Source of variation	d.f	Sum of squares (SS)	Mean SS	F-value	P-value
Genotypes	23	13,429,905	583,909	2.41	<0.001
Environments	7	54,182,532	7,740,362	31.98	<0.001
Interactions	161	38,967,120	242,032		
IPCA 1	29	21,865,873	753,996	7.81	<0.001
IPCA 2	27	6,960,918	257,812	2.67	<0.001
Residuals	105	10,140,330	96,575		

Note: The analysis was conducted using 24 genotypes evaluated across 8 environments, defined as combinations of two treatments (CV, and UC) across 4 years (2019–2022). IPCA, terms represent interaction principal components from the AMMI, model, which partition the GEI, sum of squares.

**TABLE 6:** Mean biomass yield performance (g plant^-1^) and interaction principal component axis (IPCA) scores for each environment and each genotype included in the AMMI analysis.

Environment or Genotype	S.N.	Mean	IPCA1	IPCA2
Environment				
CV19	1	2,221	−35.33	29.46
CV20	2	1895	−15.02	−13.16
CV21	3	1,587	−8.62	−22.14
CV22	4	881	−3.62	−23.03
UC19	5	1,674	−21.58	6.94
UC20	6	1942	14.39	4.64
UC21	7	2,869	36.70	22.02
UC22	8	2064	33.09	−4.72
Genotype				
J009.C	1	1868	2.77	0.94
J022.A	2	2076	5.69	9.22
J177.A	3	1800	−10.86	8.54
J188.A	4	1,374	−35.43	−2.56
J191.A	5	1,250	25.33	−15.48
J211.A	6	2081	−10.60	4.20
J218.A	7	1894	0.34	−7.32
J222.A	8	2,400	0.63	10.15
J230.A	9	1954	−5.36	−13.31
J247.A	10	1823	12.43	−13.50
J251.C	11	1914	8.02	5.09
J295.A	12	2,186	−1.97	8.47
J323.A	13	1814	6.82	2.85
J324.A	14	1966	10.65	12.55
J326.A	15	1,647	−4.12	−15.32
J456.C	16	1854	−9.22	11.26
J463.A	17	2,388	37.13	17.99
J496.C	18	2,188	−18.21	5.82
J497.C	19	1928	−10.52	0.93
J514.A	20	1,648	3.91	−17.79
J587.A	21	1887	−9.83	0.26
J587.B	22	2016	−5.50	8.72
J653.C	23	1,611	4.48	−17.49
Rambo4	24	1830	3.43	−4.24

## 4 Discussion

The positively skewed distribution of raw dry biomass yield (Supplementary Figure S2) suggests that while most genotypes exhibit low to moderate yield, certain genotypes produce exceptionally high biomass. The removal of low-yielding outliers (Supplementary Figure S3) improved the robustness of subsequent analyses, ensuring that extreme values did not disproportionately affect the results.

The results of the Shapiro-Wilk test (Table 2) confirm that the yield data were not normally distributed. The QQ plot (Supplementary Figure S4) visually reinforces this deviation, particularly at the lower and upper quantiles. This non-normality likely stems from inherent genetic variation among genotypes, environmental influences, and the distribution characteristics of biomass yield data. While statistical transformations such as log or square root transformations could potentially normalize the data, we opted to use BLUP estimates, which account for spatial and environmental variation, eliminating the need for additional transformations. This approach provides unbiased predictions for genotype performance under different environmental conditions.

To further refine yield data, a TPS regression model was applied to account for spatial variation in the field. The TPS model used spatial coordinates (row and column) as independent variables and yield values as the response variable. The residuals from the TPS model were extracted and added to the mean yield per plot to generate TPS-adjusted values. This correction ensured that spatial biases were minimized and that the final dataset more accurately represented true genetic differences among genotypes.

The repeatability estimates (Table 3) provide insights into the consistency of genetype performance for biomass yield across different environments. Notably, the higher repeatability values observed in 2019 suggest that genetic differences among genotypes were more consistently expressed under the conditions of that year. In contrast, lower repeatability values in subsequent years and under drought conditions indicate a greater influence of environmental variability on yield expression. These findings highlight the need for multi-environment trials to effectively assess genotype performance and to distinguish genetic effects from environmental noise in the evaluation of drought resilience.

The correlation analysis among drought tolerance indices provides valuable insights into their interrelationships and reliability in selecting stress-resilient genotypes (Figure 4). Across all years, MP, GMP, and STI consistently exhibited strong positive correlations, reaffirming their utility in identifying high-yielding genotypes under drought stress. In contrast, SSI was negatively correlated with resilience traits, suggesting that it effectively differentiates susceptible from tolerant genotypes. The year-to-year variability in TOL’s correlation with yield indices underscores its dependence on environmental conditions, further emphasizing the necessity of multi-year evaluations. The strong and consistent correlations among MP, GMP, and STI suggest their potential integration with genomic selection in breeding programs for drought resilience.

The temporal stability of the Drought Adaptation Index (DAI) was assessed through correlation analysis across years (Figure 5). Strong positive correlations between consecutive years indicate that DAI is a reliable index for evaluating genotypic drought response across multiple growing seasons. However, the gradual decline in correlation from 2019 to 2022 (r = 0.61) suggests that environmental variability, management practices, or GEI may have influenced drought response. These findings underscore the importance of long-term trials to ensure robust selection under diverse environmental conditions.

The DAI isoline curves provide a visual representation of yield stability across drought and control conditions (Figure 6). The classification of genotypes into very well-adapted, well-adapted, adapted, and unadapted groups enables precise differentiation of genotypic performance. The clustering of genotypes with lower BLUP values suggests that most genotypes exhibit moderate to low yield stability, while a select few demonstrate superior performance. The shifts in C1 isolines across years further reinforce the influence of environmental variability on yield stability, reinforcing the necessity for multi-year evaluations when selecting drought-resilient genotypes.

The GGE and AMMI biplots provide critical insights into GEI (Figure 7). The clustering of environments in the GGE biplot indicates distinct genotype performance patterns between drought and control treatments, with the exception of UC19, which aligned more closely with drought environments. The “Which-Won-Where” analysis identified specific genotypes, such as J222.A and J295.A, as consistently high-performing across multiple environments. The mean performance and stability biplot further supports this classification, highlighting J222.A and J295.A as stable, high-yielding genotypes suitable for breeding programs, whereas J463.A exhibited high yield potential but greater variability. The ranking of genotypes relative to the ideal genotype reinforces J222.A as a prime candidate for selection due to its balance of yield potential and stability.

The AMMI biplot analysis (Figures 14, 15; Supplementary Figure S6) further corroborates these findings, showing that genotypes with long vectors, such as J463.A exhibited strong GEI, while genotypes like J222.A and J295.A demonstrated high yield as well as greater stability across environments. The ANOVA results (Table 5) confirm that environmental effects were the dominant source of variation, followed by GEI. The high-yielding environment UC21 and the low-yielding environment CV22 highlight the impact of external factors on biomass productivity (Table 5).

This study demonstrates the utility of DAI as a robust and reliable index for selecting drought-resilient switchgrass genotypes. Compared to conventional indices, DAI offers a more comprehensive assessment of genotype performance under variable water availability conditions - drought and water available control. Additionally, our findings suggest that integrating DAI with genomic selection could further enhance the efficiency of breeding programs. Future research should explore the application of DAI in multiple locations with contrasting climatic conditions for multiple years, and investigate the physiological mechanisms underpinning drought resilience in high-performing genotypes.

While this study provides valuable insights into drought adaptation in switchgrass, several limitations should be acknowledged. First, the study was conducted in a single location, limiting its applicability to broader environmental conditions. Future research should validate the DAI across multiple geographic regions with contrasting climates to assess its robustness. Second, while BLUP-based predictions improve selection accuracy, incorporating genomic selection models could further enhance breeding efficiency by identifying molecular markers associated with drought adaptation. Third, the study focused primarily on biomass yield as an indicator of drought resilience, but additional physiological and root traits should be evaluated to gain a deeper understanding of adaptation mechanisms. Lastly, long-term studies extending beyond the four-year trial period would provide more comprehensive insights into genotype stability and environmental interactions over time.

Overall, this study provides a strong foundation for improving switchgrass adaptation to drought stress, with implications for optimizing biomass yield and stability in bioenergy and forage production systems. By integrating BLUP-based selection strategies with multi-environment evaluation metrics, we offer a robust framework for sustainable crop improvement. Further validation in diverse environments and incorporation of genomic data will enhance the applicability of DAI in accelerating breeding efforts for climate-resilient switchgrass cultivars.

## 5 Conclusion

This study introduces and validates the Drought Adaptation Index (DAI) as a novel selection tool for identifying simultaneously high yielding as well as drought-resilient switchgrass genotypes. By using BLUP predicted biomass yield under drought stressed and well-watered conditions, the DAI provides a precise and reliable method for assessing drought adaptation.

The main findings of this research include: (i) The DAI effectively differentiated switchgrass genotypes into categories of very well-adapted, well-adapted, adapted, and unadapted, providing breeders with a nuanced understanding of drought resilience across diverse genotypes, (ii) BLUP based yield estimates improved the accuracy of drought tolerance assessment by accounting for environmental and spatial variation in field trials, (iii) The comparison with multiple drought indices, including SSI, STI, GMP, and YSI, alongside the DAI, offered a comprehensive evaluation of stress tolerance mechanisms in switchgrass, (iv) Analysis of genotype stability across years provided valuable insights for breeding strategies, addressing the critical need for sustainable biomass production under variable climate conditions, and (v) The methodology developed in this study offers a robust framework for improving switchgrass adaptation to drought stress, with potential applications in breeding programs focused on enhancing both biomass yield and stress adaptation.

In summary, the DAI represents a significant advancement in switchgrass breeding strategies, offering a powerful tool for selecting genotypes that combine high yield potential with drought tolerance. This approach has broad implications for optimizing switchgrass for bioenergy production and forage applications, contributing to the development of more sustainable and climate-resilient agricultural systems. Future research should focus on validating the DAI across a wider range of environments and integrating it with genomic selection strategies to accelerate genetic gains in switchgrass improvement programs.

## Data Availability

The raw data supporting the conclusions of this article will be made available by the authors, without undue reservation.

## Supplementary material

SUPPLEMENTARY FIGURE 1 Spatial plots for each year from 2019 through 2022. CV and UC represent drought and control fields, respectively.

SUPPLEMENTARY FIGURE 2 Histogram of raw dry biomass yield (g plant^-1^) before the removal of yield data rows below 50 g plant^-1^. CV and UC represent drought and control fields, respectively.

SUPPLEMENTARY FIGURE 3 Histogram of raw dry biomass yield (g plant^-1^) after the removal of yield data rows below 50 g plant^-1^. CV and UC represent drought and control fields, respectively.

SUPPLEMENTARY FIGURE 4 QQ plot of data after outlier removal.

SUPPLEMENTARY FIGURE 5 Distribution of BLUP predicted values.

SUPPLEMENTARY FIGURE 6 Biplot of genotype mean performance (yield as BLUP-predicted value) versus second interaction principal component (IPCA2) scores.
