# Correction: Drought adaptation index (DAI) based on BLUP as a selection approach for drought-resilient switchgrass germplasm

**DOI:** 10.3389/fgene.2025.1731825

**Published:** 2025-11-12

**Authors:** Shiva Om Makaju, Hari Bahadur Chhetri, Chanaka Roshan Abeyratne, Mirko Pavicic, Hari Poudel, Jazib Ali Irfan, Anita Giabardo, Katrien M. Devos, Daniel Jacobson, Ali Mekki Missaoui

**Affiliations:** 1 Center for Applied Genetic Technologies, University of Georgia, Athens, GA, United States; 2 Institute of Plant Breeding, Genetics and Genomics, University of Georgia, Athens, GA, United States; 3 Department of Crop and Soil Sciences, University of Georgia, Athens, GA, United States; 4 Biosciences Division, Oak Ridge National Laboratory, Oak Ridge, TN, United States; 5 Agriculture and Agri-Food Canada, Lethbridge Research and Development Centre, Lethbridge, AB, Canada; 6 Department of Plant Biology, University of Georgia, Athens, GA, United States

**Keywords:** switchgrass, bioenergy, drought adaptation, yield stability, BLUP, drought adaptation index, stress tolerance indices, biomass

The following supplemental materials were omitted.

SUPPLEMENTARY FIGURE 1 | Spatial plots for each year from 2019 through 2022. CV and UC represent drought and control fields, respectively.

SUPPLEMENTARY FIGURE 2 | Histogram of raw dry biomass yield (g plant^−1^) before the removal of yield data rows below 50 g plant^−1^. CV and UC represent drought and control fields, respectively.

SUPPLEMENTARY FIGURE 3 | Histogram of raw dry biomass yield (g plant^-1^) after the removal of yield data rows below 50 g plant^-1^. CV and UC represent drought and control fields, respectively.

SUPPLEMENTARY FIGURE 4 | QQ plot of data after outlier removal.

SUPPLEMENTARY FIGURE 5 | Distribution of BLUP predicted values.

SUPPLEMENTARY FIGURE 6 | Biplot of genotype mean performance (yield as BLUP-predicted value) versus second interaction principal component (IPCA2) scores.

The file(s) have now been published.

The original article has been updated.

