# Supplementary figures and images for: Drought adaptation index (DAI) based on BLUP as a selection approach for drought-resilient switchgrass germplasm
Source: Front Genet. 2025 Aug 25;16:1626083. doi: 10.3389/fgene.2025.1626083 (PMC12414770; doi:10.3389/fgene.2025.1626083)

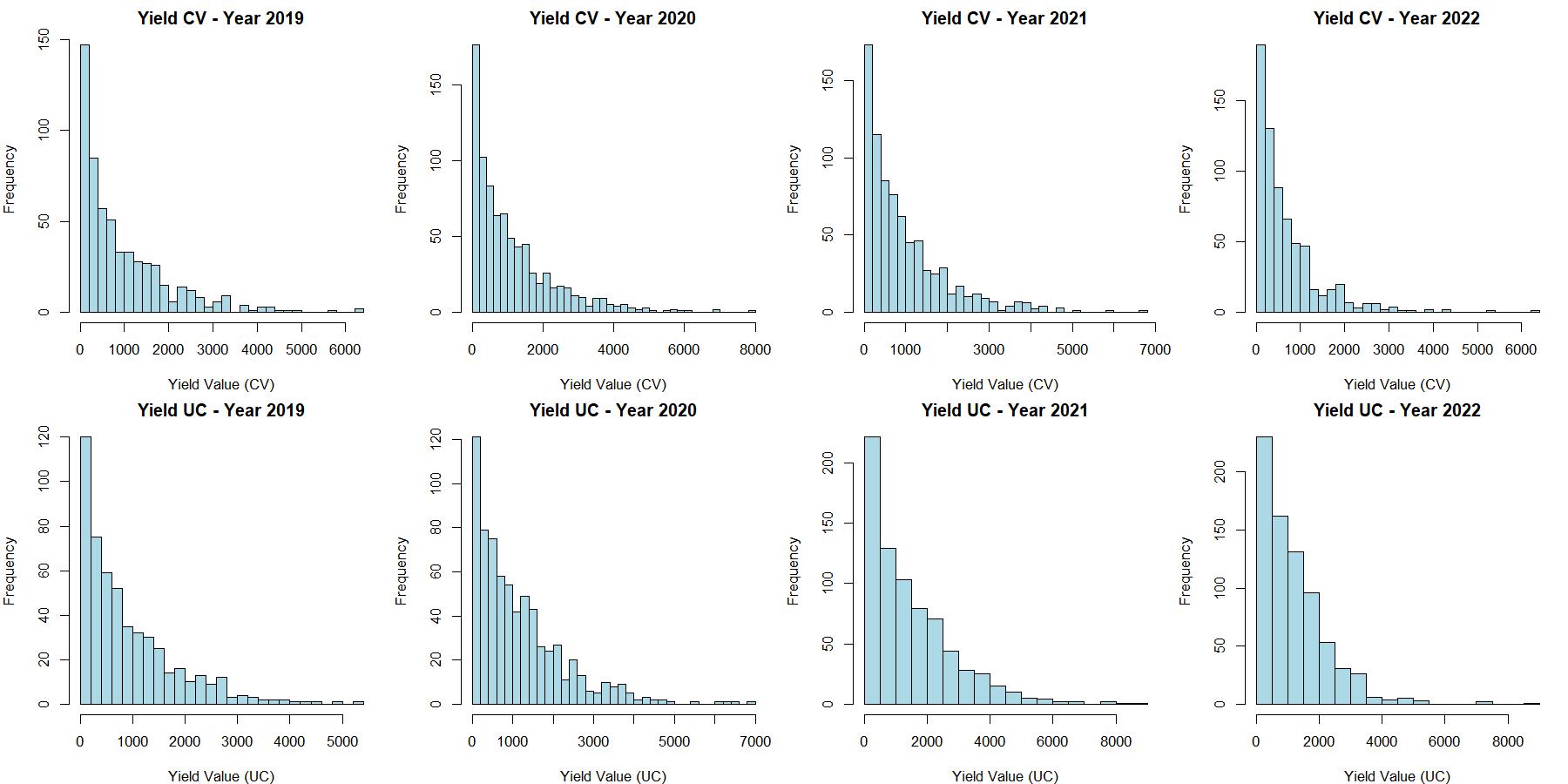

Supplement: Supplementary file 1 [file Image3.jpeg]

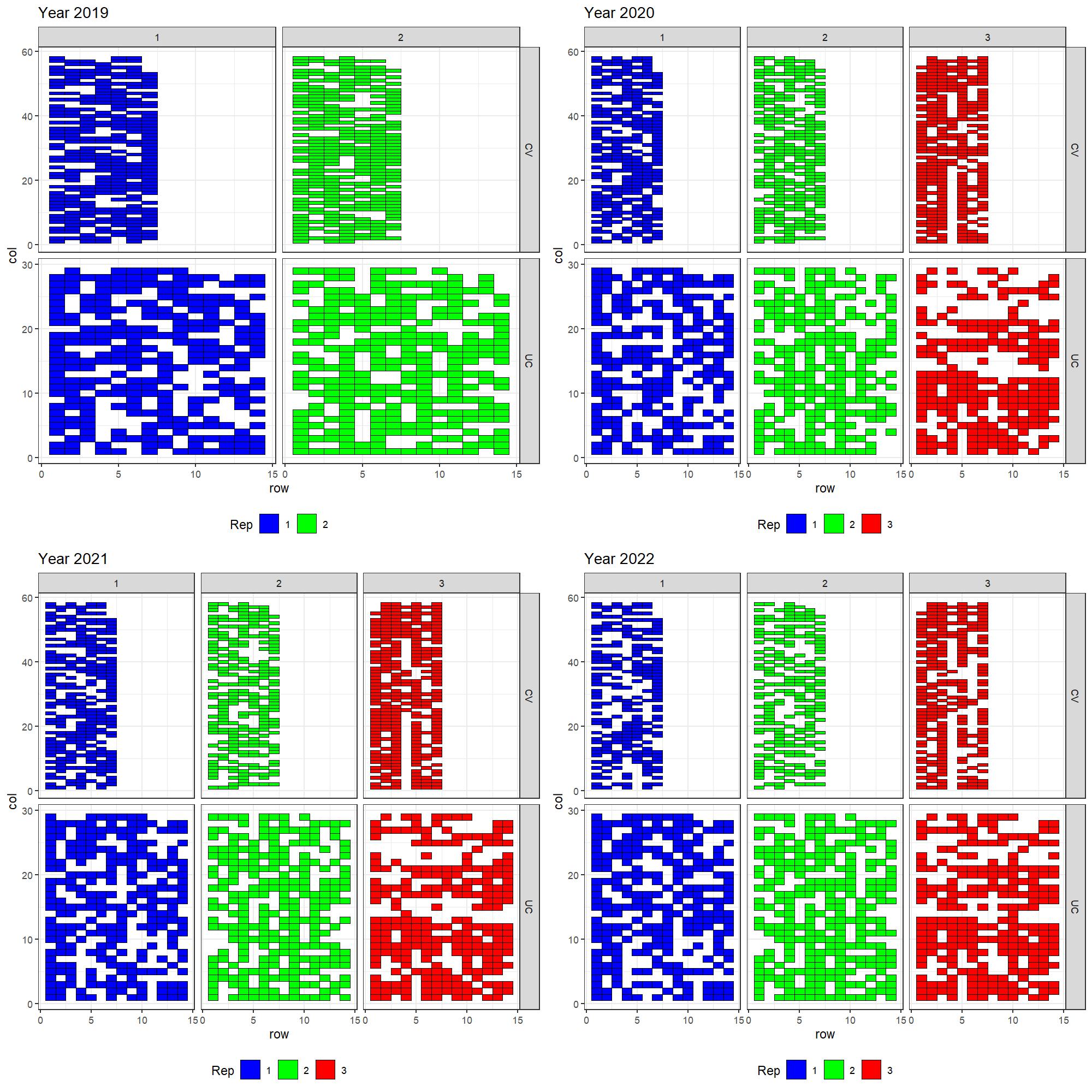

Supplement: Supplementary file 3 [file Image1.jpeg]

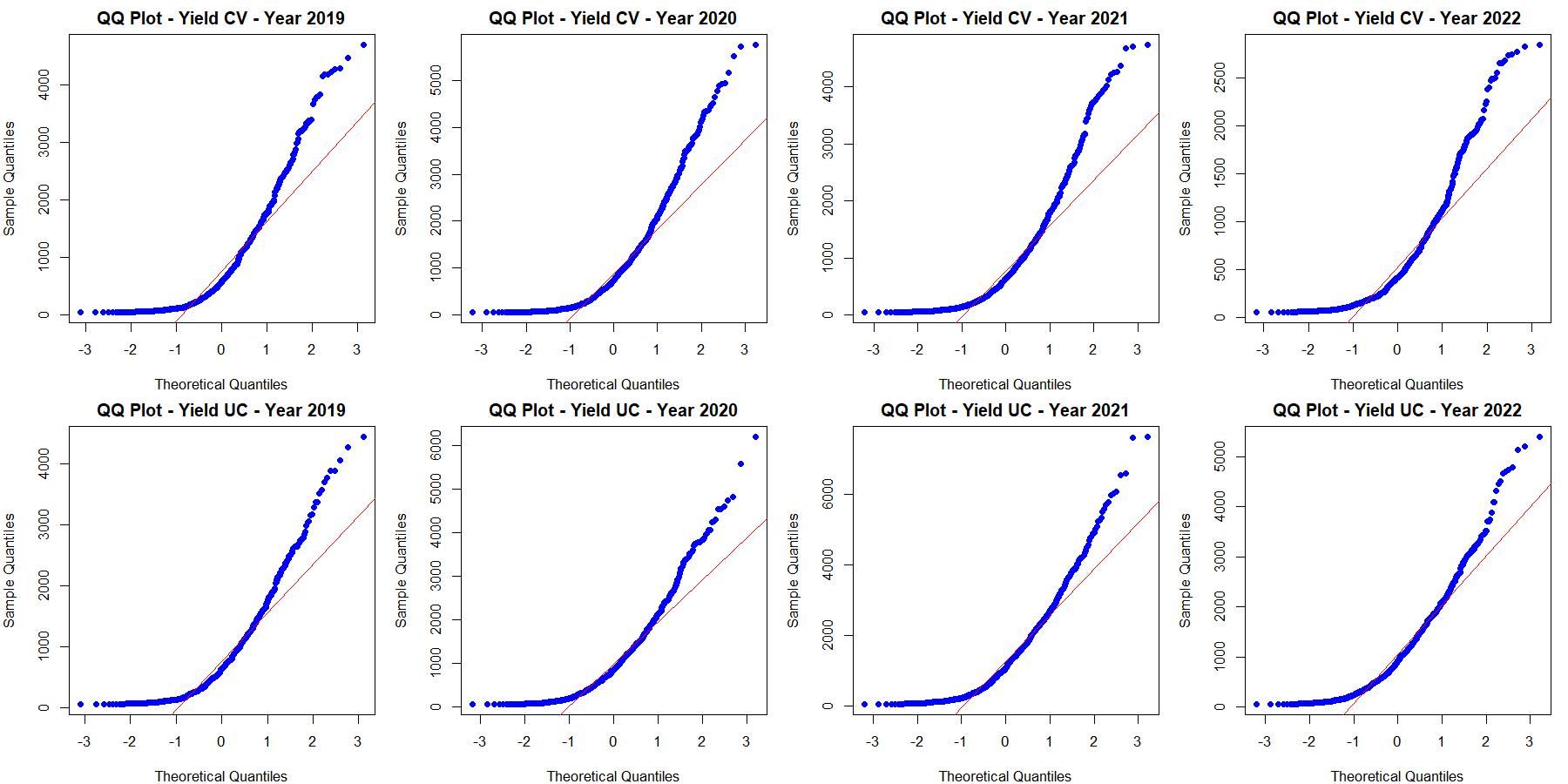

Supplement: Supplementary file 4 [file Image4.jpeg]

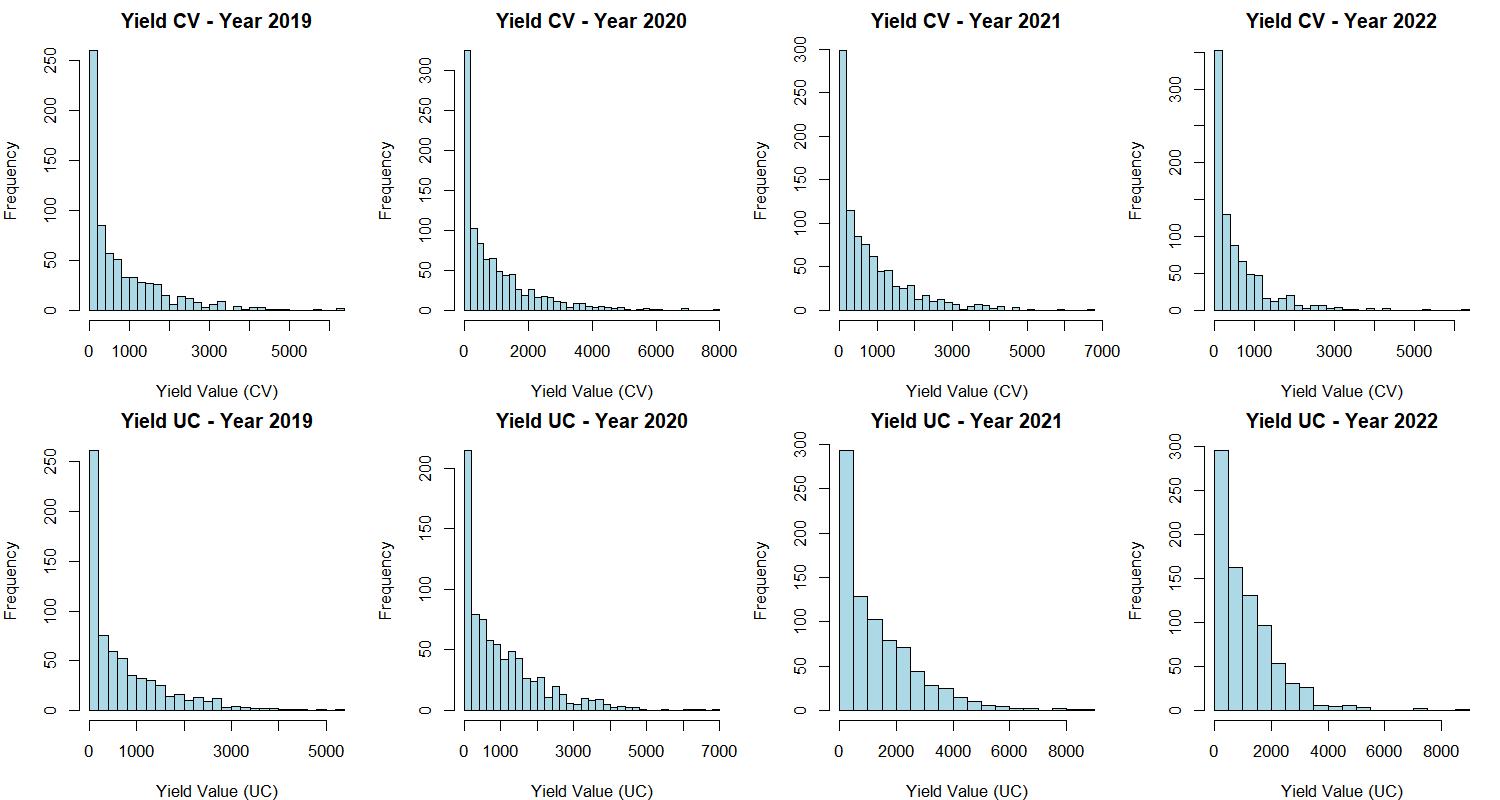

Supplement: Supplementary file 5 [file Image2.jpeg]

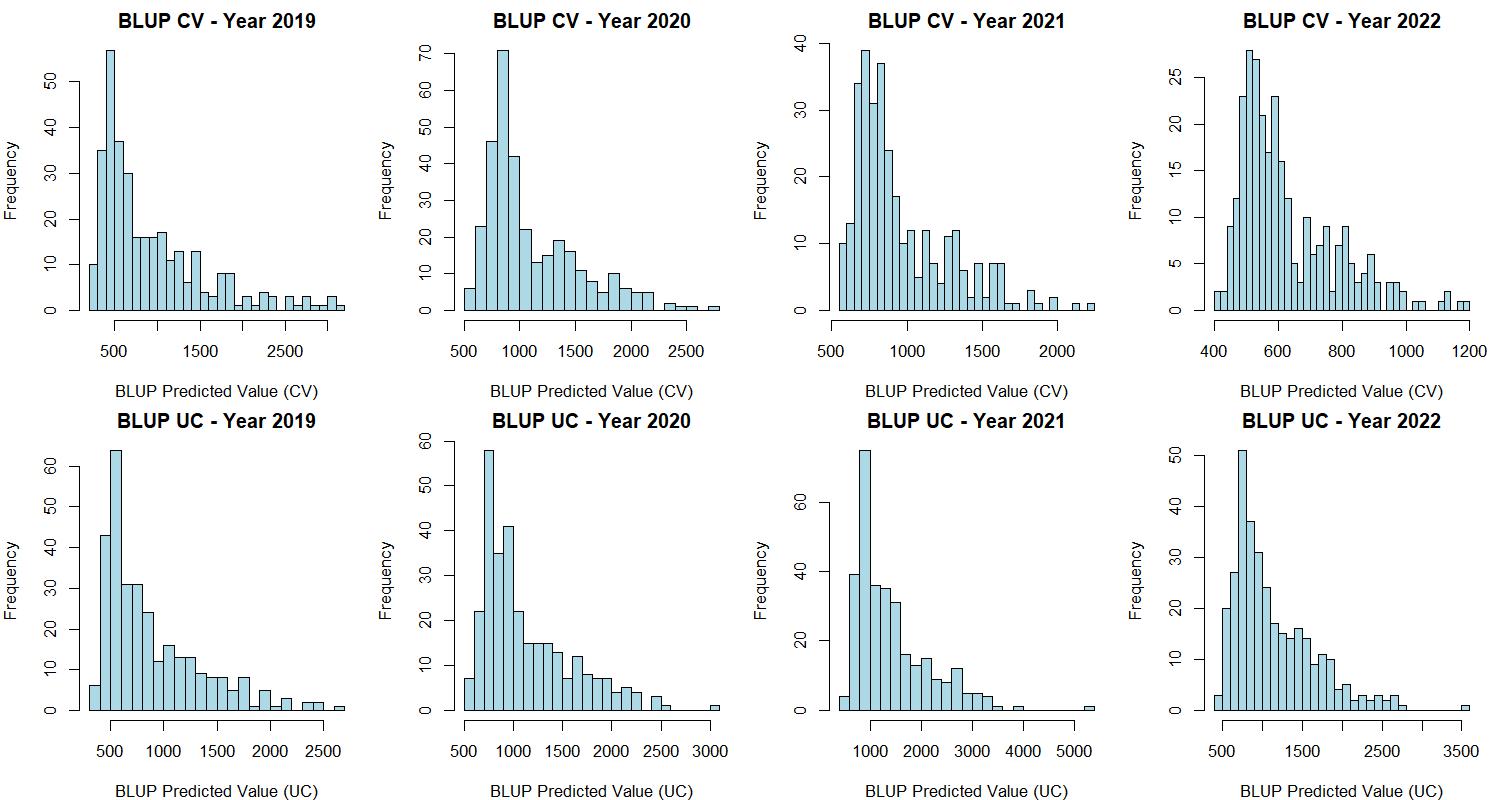

Supplement: Supplementary file 6 [file Image5.jpeg]

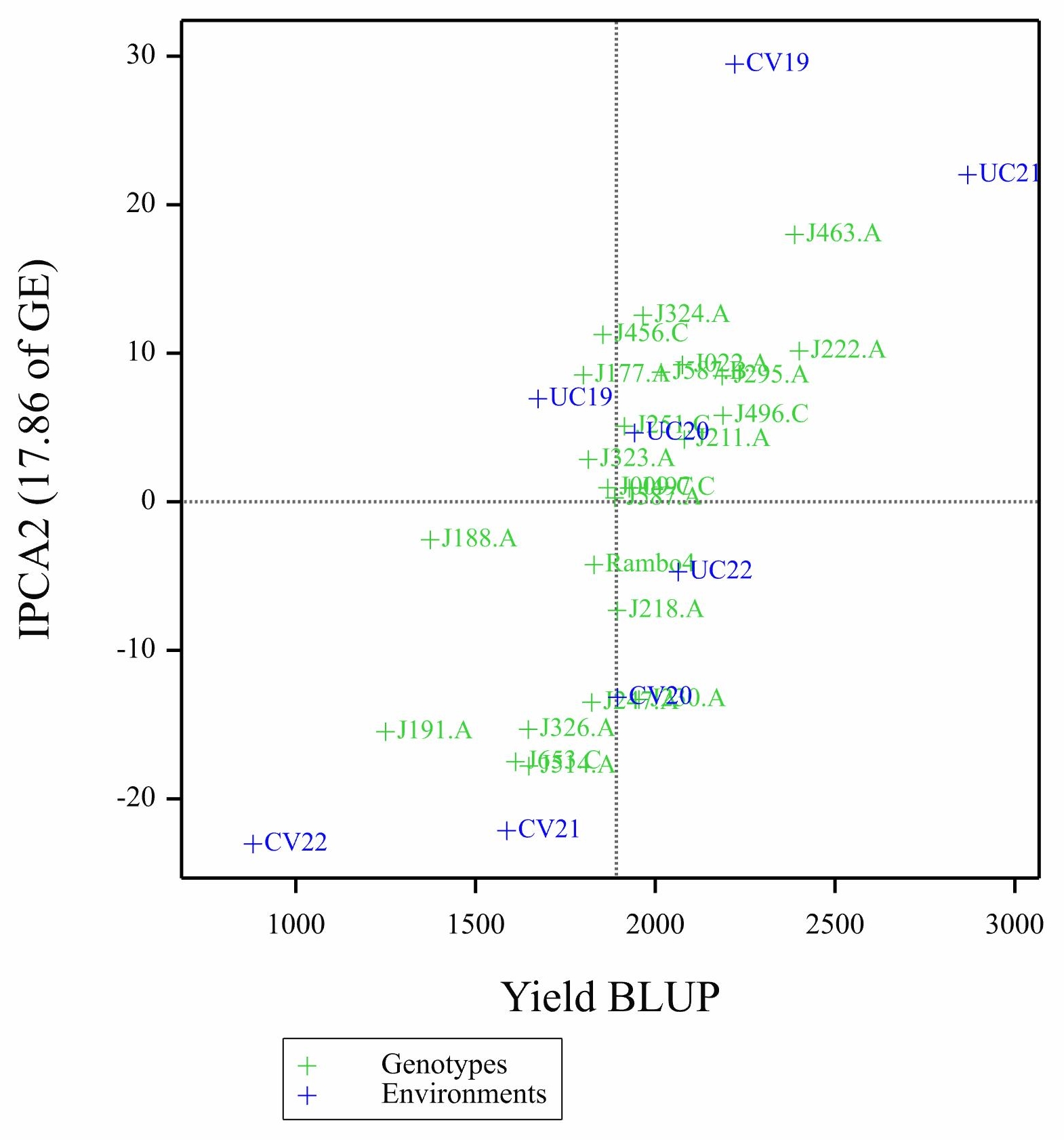

Supplement: Supplementary file 8 [file Image6.jpeg]
