# Supplementary material for: Drought adaptation index (DAI) based on BLUP as a selection approach for drought-resilient switchgrass germplasm
Source: Front Genet. 2025 Aug 25;16:1626083. doi: 10.3389/fgene.2025.1626083 (PMC12414770; doi:10.3389/fgene.2025.1626083)
Supplement: Supplementary file 2 [file Table1.docx]

| Year | Very well-adapted | Well-adapted | Adapted | Unadapted |
| --- | --- | --- | --- | --- |
| 2019 | J022.A, J177.A, J188.A, J211.A, J222.A, J295.A, J324.A, J456.C, J496.C, J497.C, J587.A, J587.B | B6, J009.C, J041.A, J218.A, J219.A, J230.A, J237.A, J240.A, J251.B, J251.C, J294.A, J305.A, J315.A, J317.A, J321.A, J323.A, J326.A, J330.A, J331.A, J463.A, J484.A, J496.A, J497.A, J587.C, Rambo10 | J001.A, J003.D, J003.E, J004.B, J008.D, J011.B, J016.A, J016.D, J019.A, J020.C, J022.B, J022.C, J023.B, J028.C, J065.A, J065.B, J073.B, J209.A, J212.A, J215.A, J226.A, J228.A, J229.A, J235.A, J236.A, J245.A, J247.A, J248.A, J249.B, J249.C, J250.A, J250.C, J251.A, J271.A, J272.A, J274.A, J275.A, J276.A, J280.A, J288.B, J296.A, J300.A, J301.A, J303.A, J308.A, J312.A, J318.A, J319.A, J320.A, J322.A, J340.A, J341.A, J419.A, J441.A, J446.B, J456.A, J456.B, J458.C, J466.A, J469.A, J469.C, J498.B, J499.A, J501.B, J514.A, J594.A, J594.C, J621.C, J652.A, Rambo4, Rambo5, TCL-7 | J001.B, J003.B, J003.C, J003.F, J005.A, J005.B, J005.D, J006.C, J008.A, J008.B, J008.C, J013.B, J013.C, J016.B, J016.C, J018.A, J018.B, J018.C, J020.B, J022.D, J026.B, J026.C, J027.B, J086.A, J086.B, J160.A, J169.A, J170.A, J175.A, J181.A, J182.A, J185.A, J186.A, J189.A, J190.A, J193.A, J194.A, J197.A, J199.A, J206.A, J208.C, J216.A, J217.A, J220.A, J223.A, J231.A, J234.A, J241.A, J243.A, J249.A, J250.B, J268.A, J270.A, J273.A, J276.B, J279.A, J293.A, J299.A, J306.B, J307.A, J311.A, J313.A, J316.A, J326.B, J327.A, J329.A, J335.A, J336.A, J337.A, J339.A, J343.A, J348.C, J349.A, J349.B, J378.C, J394.C, J416.A, J416.B, J418.A, J421.A, J424.A, J425.A, J425.B, J433.A, J441.B, J446.A, J447.A, J455.B, J458.A, J458.B, J460.A, J460.B, J460.C, J461.A, J461.C, J462.C, J464.B, J465.A, J465.B, J465.C, J466.B, J466.C, J469.B, J471.C, J481.C, J482.B, J482.C, J483.B, J483.C, J484.B, J484.C, J489.B, J491.B, J498.A, J498.C, J499.B, J499.C, J500.A, J501.A, J501.C, J502.A, J502.B, J502.C, J503.A, J503.B, J503.C, J504.C, J516.C, J522.B, J522.C, J529.C, J530.A, J533.C, J535.A, J536.A, J538.C, J540.C, J542.A, J574.B, J576.A, J576.B, J578.A, J584.A, J584.B, J584.C, J585.A, J588.B, J589.B, J590.B, J591.A, J591.B, J592.A, J593.B, J595.A, J597.B, J597.C, J599.A, J601.A, J602.A, J609.A, J610.A, J610.B, J610.C, J613.A, J614.A, J614.B, J614.C, J617.A, J621.A, J645.C, J653.C, J681.A, TCL-32 |
| 2020 | J211.A, J218.A, J222.A, J230.A, J295.A, J463.A, J496.C | J004.B, J009.C, J022.A, J177.A, J215.A, J219.A, J228.A, J237.A, J247.A, J249.C, J251.A, J251.C, J280.A, J294.A, J317.A, J321.A, J323.A, J326.A, J330.A, J331.A, J456.C, J484.A, J496.A, J497.A, J497.C, J514.A, J587.A, J587.B, J587.C, J594.C, J653.C, Rambo4, Rambo5 | B6, J003.D, J005.A, J008.D, J011.B, J016.A, J016.D, J019.A, J020.C, J022.B, J022.C, J022.D, J023.B, J028.C, J030.C, J041.A, J065.A, J065.B, J073.A, J073.B, J173.A, J199.A, J209.A, J212.A, J220.A, J226.A, J229.A, J235.A, J245.A, J249.B, J250.C, J251.B, J268.A, J271.A, J274.A, J275.A, J276.A, J276.B, J293.A, J296.A, J300.A, J301.A, J305.A, J308.A, J312.A, J315.A, J319.A, J322.A, J324.A, J326.B, J340.A, J341.A, J419.A, J441.A, J456.A, J458.A, J460.A, J469.A, J469.C, J484.C, J498.B, J499.A, J499.B, J499.C, J501.B, J521.B, J594.A, J610.B, J615.A, Rambo10, TCL-7 | J001.A, J003.B, J003.E, J003.F, J005.B, J005.D, J008.A, J008.B, J008.C, J013.B, J013.C, J016.B, J016.C, J018.A, J018.B, J018.C, J020.B, J026.B, J026.C, J027.B, J086.A, J086.B, J169.A, J181.A, J182.A, J185.A, J186.A, J188.A, J189.A, J190.A, J191.A, J193.A, J194.A, J197.A, J206.A, J208.B, J208.C, J210.A, J216.A, J217.A, J223.A, J231.A, J234.A, J236.A, J240.A, J241.A, J243.A, J246.A, J248.A, J249.A, J250.A, J250.B, J270.A, J272.A, J273.A, J279.A, J288.B, J297.A, J299.A, J303.A, J306.B, J306.C, J307.A, J313.A, J316.A, J318.A, J320.A, J327.A, J329.A, J335.A, J336.A, J337.A, J339.A, J348.C, J394.C, J416.A, J418.A, J419.B, J422.A, J424.A, J425.A, J425.B, J441.B, J446.A, J446.B, J447.A, J455.B, J456.B, J458.B, J458.C, J460.B, J460.C, J461.A, J461.C, J462.C, J464.B, J465.A, J465.B, J465.C, J466.A, J466.B, J466.C, J466.D, J469.B, J477.B, J482.B, J482.C, J483.A, J483.B, J483.C, J484.B, J497.B, J498.A, J498.C, J500.A, J500.C, J501.A, J501.C, J502.A, J502.B, J502.C, J503.A, J503.B, J503.C, J504.A, J504.C, J516.C, J517.C, J522.C, J535.A, J536.A, J540.B, J574.B, J576.A, J576.B, J578.A, J584.A, J584.B, J584.C, J585.A, J585.B, J586.A, J588.B, J589.B, J591.A, J591.B, J592.A, J593.B, J595.C, J596.A, J597.A, J597.B, J597.C, J599.A, J599.B, J601.A, J602.A, J609.A, J610.A, J610.C, J612.C, J613.A, J613.B, J621.A, J621.C, J645.C, J652.A, J652.C, TCL-32 |
| 2021 | J009.C, J222.A, J230.A, J247.A, J295.A, J324.A, J463.A, J496.C, J587.B, J653.C, Rambo4 | J004.B, J011.B, J016.A, J022.A, J022.C, J065.A, J211.A, J215.A, J218.A, J220.A, J249.C, J251.A, J251.C, J274.A, J280.A, J315.A, J317.A, J319.A, J321.A, J323.A, J326.A, J330.A, J456.C, J497.C, J499.B, J514.A, J587.A, J587.C, J594.C, Rambo10, Rambo5 | J001.A, J003.D, J008.A, J008.D, J016.D, J020.C, J022.B, J022.D, J023.B, J028.C, J041.A, J065.B, J073.A, J073.B, J177.A, J188.A, J191.A, J199.A, J209.A, J219.A, J226.A, J228.A, J237.A, J240.A, J245.A, J249.B, J250.B, J250.C, J251.B, J268.A, J271.A, J272.A, J276.B, J294.A, J296.A, J300.A, J301.A, J305.A, J308.A, J312.A, J318.A, J322.A, J326.B, J331.A, J340.A, J341.A, J419.A, J419.B, J441.A, J456.A, J458.A, J469.A, J484.A, J484.C, J496.A, J497.A, J498.B, J499.A, J501.B, J584.A, J584.C, J594.A, J610.B, J652.A | B6, J001.B, J003.B, J003.C, J003.E, J003.F, J005.A, J005.B, J005.D, J008.B, J008.C, J013.B, J013.C, J016.B, J016.C, J018.A, J018.B, J018.C, J019.A, J020.B, J026.B, J026.C, J027.B, J086.A, J086.B, J169.A, J173.A, J181.A, J182.A, J186.A, J189.A, J190.A, J193.A, J194.A, J197.A, J206.A, J208.B, J208.C, J210.A, J212.A, J216.A, J217.A, J223.A, J229.A, J231.A, J234.A, J235.A, J236.A, J241.A, J243.A, J246.A, J248.A, J249.A, J250.A, J270.A, J273.A, J275.A, J276.A, J279.A, J288.B, J293.A, J297.A, J299.A, J303.A, J306.B, J306.C, J307.A, J313.A, J314.A, J316.A, J320.A, J327.A, J329.A, J335.A, J336.A, J337.A, J339.A, J343.A, J374.A, J394.C, J416.A, J418.A, J422.A, J424.A, J441.B, J446.A, J446.B, J447.A, J455.B, J456.B, J458.B, J458.C, J460.A, J460.B, J460.C, J461.A, J461.C, J462.C, J465.A, J465.B, J465.C, J466.A, J466.B, J466.D, J469.B, J469.C, J477.B, J482.B, J482.C, J483.A, J483.B, J483.C, J484.B, J497.B, J498.A, J498.C, J499.C, J500.A, J500.C, J501.A, J501.C, J502.A, J502.B, J502.C, J503.A, J503.B, J503.C, J504.A, J504.C, J513.A, J516.C, J517.C, J522.B, J522.C, J529.C, J535.A, J540.B, J541.B, J574.B, J576.A, J576.B, J578.A, J584.B, J585.A, J585.B, J586.A, J588.B, J589.B, J590.B, J591.B, J593.B, J595.A, J595.C, J596.A, J597.A, J597.B, J597.C, J599.A, J599.B, J601.A, J602.A, J609.A, J610.A, J610.C, J613.A, J613.B, J614.A, J614.B, J614.C, J621.A, J621.C, J645.C, TCL-32, TCL-7 |
| 2022 | J191.A, J222.A, J247.A, J295.A, J463.A, J514.A | J009.C, J016.A, J022.A, J065.A, J173.A, J211.A, J215.A, J218.A, J219.A, J220.A, J230.A, J249.C, J251.C, J280.A, J308.A, J315.A, J318.A, J319.A, J323.A, J324.A, J326.A, J496.C, J497.C, J499.B, J587.A, J587.B, J594.C, J653.C, Rambo10, Rambo4, Rambo5 | J001.A, J003.D, J004.B, J008.A, J008.D, J011.B, J016.B, J016.D, J020.C, J022.B, J022.C, J022.D, J023.B, J028.C, J065.B, J073.A, J177.A, J199.A, J209.A, J216.A, J223.A, J226.A, J228.A, J237.A, J240.A, J241.A, J245.A, J249.B, J250.B, J250.C, J251.A, J251.B, J268.A, J271.A, J272.A, J273.A, J274.A, J276.B, J279.A, J293.A, J294.A, J301.A, J305.A, J312.A, J317.A, J321.A, J322.A, J326.B, J330.A, J340.A, J341.A, J394.C, J419.A, J419.B, J441.B, J456.A, J456.C, J458.A, J469.A, J484.A, J484.C, J496.A, J497.A, J498.B, J499.A, J502.A, J516.C, J587.C, J596.A, J610.B, J610.C | B6, J001.B, J003.B, J003.E, J005.A, J005.D, J008.B, J008.C, J013.B, J013.C, J016.C, J018.A, J018.B, J018.C, J019.A, J020.B, J026.B, J027.B, J041.A, J073.B, J086.A, J086.B, J169.A, J175.A, J181.A, J182.A, J186.A, J188.A, J189.A, J190.A, J193.A, J197.A, J206.A, J208.C, J210.A, J212.A, J217.A, J229.A, J231.A, J234.A, J235.A, J236.A, J243.A, J246.A, J248.A, J249.A, J250.A, J270.A, J275.A, J276.A, J288.B, J296.A, J297.A, J299.A, J300.A, J303.A, J306.B, J306.C, J307.A, J313.A, J316.A, J320.A, J327.A, J329.A, J331.A, J335.A, J336.A, J337.A, J339.A, J343.A, J348.C, J374.A, J396.A, J416.A, J418.A, J424.A, J441.A, J446.A, J446.B, J447.A, J456.B, J458.B, J458.C, J460.A, J460.B, J460.C, J461.A, J462.C, J465.A, J465.B, J465.C, J466.A, J466.B, J466.D, J469.B, J469.C, J477.B, J482.B, J482.C, J483.A, J483.C, J484.B, J497.B, J498.A, J498.C, J499.C, J500.A, J500.C, J501.A, J501.B, J501.C, J502.B, J502.C, J503.A, J503.B, J503.C, J504.A, J504.C, J517.C, J535.A, J536.B, J576.A, J576.B, J578.A, J584.A, J584.B, J584.C, J585.B, J586.A, J588.B, J590.B, J591.B, J593.B, J594.A, J595.A, J595.C, J597.A, J597.B, J597.C, J599.A, J601.A, J602.A, J610.A, J612.C, J613.A, J614.A, J614.B, J614.C, J619.A, J621.A, J621.C, J645.C, J652.A, TCL-32, TCL-7 |
