# Supplementary material for: Drought adaptation index (DAI) based on BLUP as a selection approach for drought-resilient switchgrass germplasm
Source: Front Genet. 2025 Aug 25;16:1626083. doi: 10.3389/fgene.2025.1626083 (PMC12414770; doi:10.3389/fgene.2025.1626083)
Supplement: Supplementary file 7 [file Table2.docx]

**Supplementary Table 2. Monthly Averages of Daily Maximum (Max.) and Minimum (Min.) Temperatures and Total Precipitation in Tifton, Georgia (2019–2022)**

|  | **Mean Daily Max. Temperature (^o^C)** | | | |  | **Mean Daily Min. Temperature (^o^C)** | | | |  | **Monthly Precipitation (cm)** | | | |
| --- | --- | --- | --- | --- | --- | --- | --- | --- | --- | --- | --- | --- | --- | --- |
| **Month** | **2019** | **2020** | **2021** | **2022** |  | **2019** | **2020** | **2021** | **2022** |  | **2019** | **2020** | **2021** | **2022** |
| January | 16 | 17.4 | 15.1 | 15.5 |  | 5.1 | 7.2 | 5.7 | 3.7 |  | 10 | 8.2 | 17.9 | 16.9 |
| February | 20.7 | 18.3 | 17.1 | 19.6 |  | 10.3 | 7.3 | 6.4 | 7.2 |  | 5.4 | 15.4 | 22.4 | 6.1 |
| March | 20.8 | 24.7 | 23.3 | 23.7 |  | 9 | 13.7 | 11.1 | 10.5 |  | 10 | 12.6 | 11.8 | 10.1 |
| April | 25.9 | 25.3 | 24.2 | 25.5 |  | 13.2 | 12.9 | 12 | 12.7 |  | 6.1 | 14.5 | 17.4 | 6 |
| May | 32 | 28.3 | 28.7 | 30.4 |  | 19.5 | 16.3 | 15.7 | 18 |  | 4.9 | 6.6 | 2.6 | 3.2 |
| June | 32.1 | 30.8 | 31.3 | 34.2 |  | 21.4 | 20.8 | 21.1 | 21.8 |  | 14.3 | 12.9 | 20.2 | 9.7 |
| July | 33.6 | 33.7 | 31.7 | 32.9 |  | 22.1 | 22.3 | 22 | 22.4 |  | 4.9 | 4.7 | 20.5 | 14.4 |
| August | 34.5 | 33.3 | 32 | 32.3 |  | 22.3 | 22.5 | 22.5 | 21.9 |  | 16.2 | 11.4 | 14.9 | 20.6 |
| September | 34.9 | 29.4 | 30.4 | 29.6 |  | 20.3 | 20.1 | 19.2 | 18.6 |  | 1.1 | 13.3 | 9 | 6.3 |
| October | 28.1 | 27.7 | 26.5 | 25.1 |  | 16.9 | 16.8 | 15.1 | 10.7 |  | 10.8 | 6.2 | 9.3 | 2.6 |
| November | 19.2 | 23.1 | 20.1 | 20.8 |  | 7.4 | 12.4 | 6.2 | 10.4 |  | 3.8 | 6.3 | 1.3 | 8.6 |
| December | 17.4 | 15.8 | 21.2 | 16.7 |  | 7.5 | 3.6 | 10.5 | 6 |  | 13.3 | 12.1 | 7 | 3.8 |

**Note:** Weather data were obtained from the University of Georgia Weather Network (2025); see References.
